# Empowering Carbon Fibers With Ti_3_C_2_T_x_ MXene: A Paradigm Shift Toward Integrated Structure‐Function Composites

**DOI:** 10.1002/advs.202524225

**Published:** 2026-02-05

**Authors:** Hongshuo Cao, Yue Xing, Jiangman Sun, Yanhong Tian, Yangyang Gao, Xuejun Zhang, Xiubing Liang

**Affiliations:** ^1^ State Key Laboratory of Organic‐Inorganic Composites Beijing University of Chemical Technology Beijing China; ^2^ National Innovation Institute of Defense Technology Academy of Military Science PLA China Beijing China; ^3^ Ocean College Zhejiang University Zhoushan China

**Keywords:** carbon fiber, electromagnetic interference shielding, modification strategies, multifunctional applications, smart sensing materials, Ti_3_C_2_T_x_ MXene

## Abstract

This review delineates a transformative strategy in advanced materials: the integration of Ti_3_C_2_T_x_ MXene with carbon fibers (CFs) to forge a new class of multifunctional structural composites. This integration strategy signifies a paradigm shift from simple structural components to multifunctional material systems. Moving beyond conventional interface enhancement, precise modification techniques such as self‐assembly, electrophoretic deposition, chemical grafting, and blending‐spinning synergistically combine the outstanding mechanical properties of CFs with the diverse electrical, thermal, and optical characteristics of Ti_3_C_2_T_x_ MXene. This synergistic coupling effectively overcomes the long‐standing limitations of CFs, including surface inertness and functional singularity. The review systematically examines the resulting performance improvements across a range of frontier applications, including interface reinforcement, electromagnetic shielding, battery energy storage, smart sensing, and thermal management. However, achieving industrial applications still depends on overcoming key challenges related to Ti_3_C_2_T_x_ MXene stability, scalable processing, and multifunctional optimization. This review not only summarizes current research progress but also outlines a roadmap for future studies, emphasizing sustainable processing, interfacial nanoengineering, and the rational design of next‐generation structure‐function‐integrated composites.

## Graphical abstract

This review comprehensively outlines how Ti_3_C_2_T_x_ MXene transforms carbon fiber from a structural component into a multifunctional platform. We systematically detail cutting‐edge modification strategies and showcase exceptional performance in EMI shielding, energy storage, smart sensing, and beyond. The article critically addresses key challenges and charts a course for the future development of these structurally integrated, next‐generation materials.

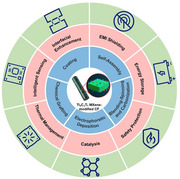

## 1 Introduction

Since its emergence in the 1960s, carbon fiber (CF) has experienced rapid industrial growth. With continuous improvements in manufacturing processes, CFs have quickly established a leading position in the fiber market owing to their outstanding specific strength, specific modulus, excellent corrosion resistance, high electrical conductivity, and thermal conductivity [1, 2]. Over the past few years, advances in quality and large‐scale production have broadened the applications of CFs to include aerospace, transportation, military industries, wind power, and civilian markets, signifying their vital importance in modern society [3, 4, 5, 6].

As application scenarios for CFs continue to expand, researchers are no longer content to use CFs solely as reinforcement agents in composites. Harnessing their exceptional electrical and thermal properties, extensive research efforts have been devoted in recent years to exploring their functional applications [7, 8]. However, the predominance of non‐active carbon on the surface of CFs results in highly inert surfaces with extremely low interfacial interaction and reactivity, which greatly limits the realization of their multifaceted performance advantages [9, 10]. Modification has proven to be an effective means of overcoming these limitations. Modified CFs not only exhibit enhanced surface activity and interfacial interactions, but also unlock the functional advantages of the fibers themselves, opening up new possibilities for constructing ideal multifunctional structural materials [11, 12, 13].

More recently, 2D nanomaterials such as graphene, carbon nanotubes (CNTs), and MXenes have undoubtedly infused new vitality into the modification of CFs [14, 15, 16, 17]. The excellent mechanical properties and high specific surface area of 2D nanomaterials offer efficient strategies for constructing multiscale interfacial layers in CF reinforced polymer (CFRP), while their metallic‐like conductivity, thermal conductivity, and unique structures open up new avenues for functional applications of CFs.

Among numerous 2D nanomaterials, MXene stands out with significant advantages. Since its discovery in 2011, MXene has become one of the most promising research frontiers in materials science, including for the modification of CFs and their composites [18, 19]. MXenes are composed of transition metal carbides, nitrides, or carbonitrides, with the general formula M_n+1_X_n_T_x_ (where M represents a transition metal, X is C or N, T refers to surface terminations such as ─OH, ─O, and ─F, n typically ranges from 1 to 3), of these, Ti_3_C_2_T_x_ MXene is by far the most studied and utilized [20, 21]. As the first discovered MXene, it is typically obtained by selectively etching the MAX phase precursor (Ti_3_AlC_2_) with an etchant. Its surface is abundant with active functional groups, thereby granting it remarkable hydrophilicity (with a contact angle <20°), chemical reactivity, and terminal‐group tunability, these properties render it an exceptionally processable 2D nanomaterial via solution‐based methods, outperforming surface‐inert materials like pristine graphene (>90°) and CNTs (>100°) [22, 23]. Remarkably, among numerous solution‐processable 2D nanomaterials, monolayer Ti_3_C_2_T_x_ MXene exhibits a Young's modulus exceeding 330 GPa, demonstrating outstanding mechanical properties that are notably higher than those of graphene oxide (∼200 GPa) and reduced graphene oxide (∼250 GPa) [24]. This makes it highly valuable for applications such as interfacial reinforcement in CFRPs. The electrical performance of Ti_3_C_2_T_x_ MXene is also particularly noteworthy. It has been reported that Ti_3_C_2_T_x_ MXene can achieve a volumetric capacitance of up to 676 F·cm^−3^, significantly exceeding that of other 2D materials such as graphene (∼30 F·cm^−3^) and CNTs (∼50 F·cm^−3^), highlighting its exceptional electrical properties [25]. This characteristic highlights its significant potential in electromagnetic interference (EMI) shielding, energy storage, smart sensing, and related fields. Additionally, Ti_3_C_2_T_x_ MXene demonstrates excellent thermal conductivity; for example, the addition of only 0.3 wt% Ti_3_C_2_T_x_ MXene notably increases the thermal conductivity of paraffin by 16%, suggesting broad prospects in thermal management and related areas [26]. Owing to these superior comprehensive properties, the use of Ti_3_C_2_T_x_ MXene for CF modification has garnered significant attention and achieved remarkable progress in recent years.

**FIGURE 1:**
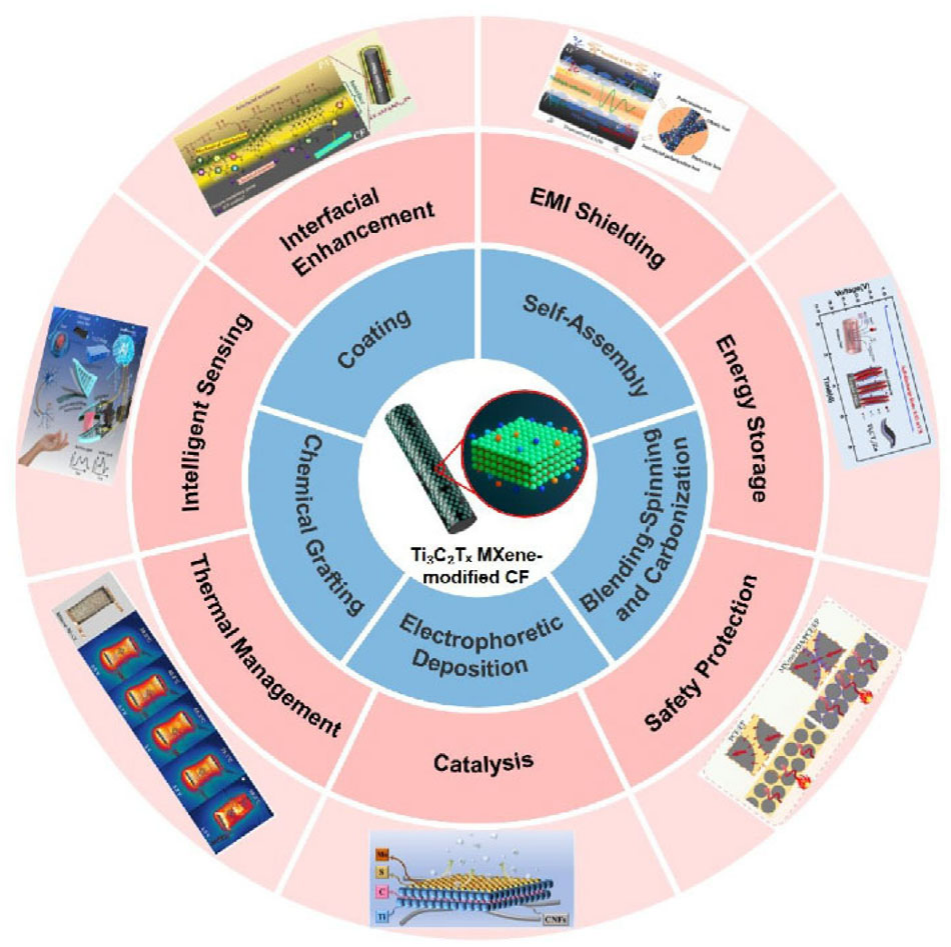
Schematic illustration showing the principal fabrication methods of Ti_3_C_2_T_x_ MXene‐modified CF and representative studies in key application areas. Figures related to the key application areas are reproduced with permission as follows: Interfacial enhancement; Reproduced with permission [35]. Copyright 2024, American Chemical Society. EMI shielding; Reproduced with permission [36]. Copyright 2025, Elsevier. Energy storage; Reproduced with permission [37]. Copyright 2022, American Chemical Society. Safety protection; Reproduced with permission [38]. Copyright 2025, Wiley. Catalysis; Reproduced with permission [39]. Copyright 2022, Elsevier. Thermal management; Reproduced under terms of the CC‐BY license [40]. Copyright 2023, Elsevier. Smart sensing; Reproduced with permission [41]. Copyright 2025, Wiley.

Over time, the application of Ti_3_C_2_T_x_ MXene in CF modification has evolved considerably. Figure 1 illustrates the principal fabrication strategies of Ti_3_C_2_T_x_ MXene‐modified CF and highlights representative studies in the key application domains. With the introduction of techniques such as electrophoretic deposition (EPD) and electrostatic self‐assembly, the modification process of Ti_3_C_2_T_x_ MXene on CF has entered a new era characterized by simplicity, rapidity, uniformity, and control [27, 28, 29]. These advances offer new possibilities for large‐scale, continuous production and for the controlled design of CF surfaces. On this basis, synergistic design and further functionalization of Ti_3_C_2_T_x_ MXene have been employed to tailor Ti_3_C_2_T_x_ MXene‐modified CF for a wider range of applications, greatly enhancing the practical performance and application potential in various areas [30]. Exploiting the combined mechanical, electrical, and thermal properties of Ti_3_C_2_T_x_ MXene and CF, Ti_3_C_2_T_x_ MXene‐modified CFs have found increasing use in interfacial strengthening, battery energy storage, EMI shielding, thermal management, and smart sensing, with their popularity and innovation continuing to grow [31, 32]. Given the outstanding promise of Ti_3_C_2_T_x_ MXene‐modified CFs, further research in this area is likely to have a pivotal influence on numerous interdisciplinary fields. However, despite significant progress, several challenges remain in this field. One key issue is the relatively complex preparation process of Ti_3_C_2_T_x_ MXene, as well as its susceptibility to oxidative degradation [33, 34]. These factors pose challenges to the effective service life of Ti_3_C_2_T_x_ MXene‐modified CFs, emphasizing the urgency of systematic and targeted research efforts.

This review systematically summarizes recent advances in the preparation of Ti_3_C_2_T_x_ MXene‐modified CFs, providing a technical reference for their fabrication. As research on Ti_3_C_2_T_x_ MXene‐modified CFs expands, this review also broadly highlights recent progress and applications in various fields, which is critical for fully realizing the potential of Ti_3_C_2_T_x_ MXene‐modified CFs. Finally, the review discusses current challenges and future prospects, aiming to guide the optimization of modification strategies and the expansion of application domains of Ti_3_C_2_T_x_ MXene‐modified CFs.

## 2 Preparation of Ti_3_C_2_T_x_ MXene‐Modified CFs

The selection of preparation methods for Ti_3_C_2_T_x_ MXene‐modified CFs is determined not only by the intrinsic properties of the materials and their intended applications, but also by external considerations such as cost, energy consumption, and scalability. An optimal synthesis strategy provides the foundation for harnessing the unique functionalities of Ti_3_C_2_T_x_ MXene‐modified CFs across different application domains. It should be noted that, prior to Ti_3_C_2_T_x_ MXene modification, CFs are typically subjected to oxidative or carboxylative pretreatments to introduce ‐OH and ‐COOH functional groups, facilitating improved adhesion to the Ti_3_C_2_T_x_ MXene. Unless otherwise specified, all CFs discussed in this review have undergone such pretreatments.

### 2.1 Coating Techniques

Coating is a traditional and widely used approach for CF modification. This method involves immersion or application of the modifying agent onto the CF surface, resulting in effective encapsulation or surface‐layer formation; such strategies are readily adaptable for Ti_3_C_2_T_x_ MXene‐functionalization of CFs [42, 43]. Studies have shown that Ti_3_C_2_T_x_ MXene exhibits excellent hydrophilicity and good colloidal stability, with a Zeta potential in water ranging from −30 to −60 mV [20]. This indicates that Ti_3_C_2_T_x_ MXene can form stable colloidal dispersions, making it an effective sizing agent for CFs. Additionally, the abundant active functional groups on Ti_3_C_2_T_x_ MXene facilitate stronger interactions such as hydrogen bonding and van der Waals forces, with pretreated CF surfaces [43]. The simplicity, low cost, and energy efficiency of the coating method have facilitated its widespread adoption for Ti_3_C_2_T_x_ MXene‐CF modification.

**FIGURE 2:**
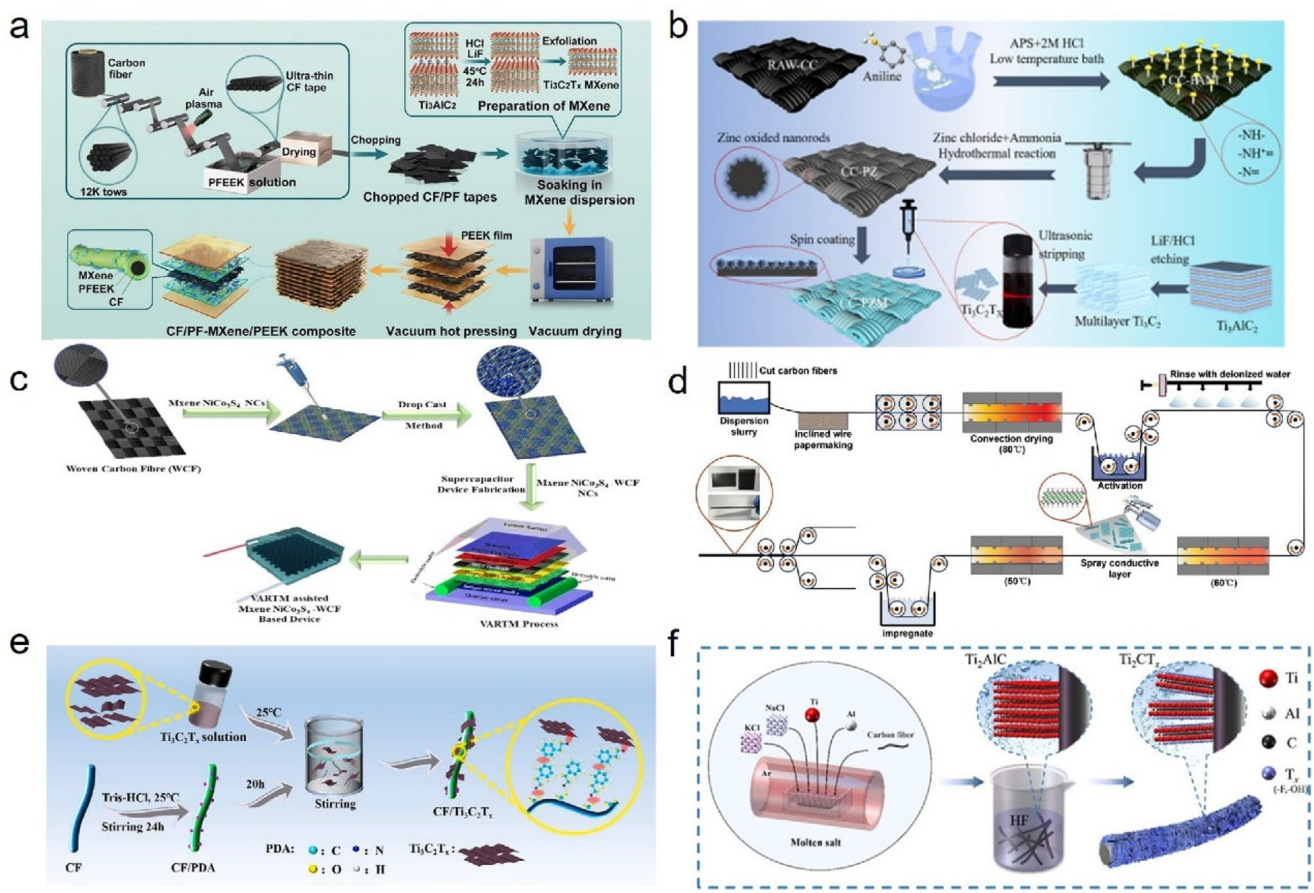
(a) Schematic diagram of the dip‐coating‐based preparation of Ti_3_C_2_T_x_ MXene‐modified CF; Reproduced with permission [45]. Copyright 2024, Elsevier. (b) Schematic diagram of the spin‐coating‐based preparation of Ti_3_C_2_T_x_ MXene‐modified CF; Reproduced with permission [46]. Copyright 2025, Wiley. (c) Schematic diagram of the drop‐casting‐based preparation of Ti_3_C_2_T_x_ MXene‐modified CF; Reproduced with permission [47], Copyright 2024, Elsevier. (d) Schematic diagram of the spray‐coating‐based preparation of Ti_3_C_2_T_x_ MXene‐modified CF; Reproduced with permission [16]. Copyright 2025, Wiley. (e) Schematic illustration of the PDA‐assisted coating process for fabricating Ti_3_C_2_T_x_ MXene‐modified CF; Reproduced with permission [50]. Copyright 2022, Elsevier. (f) Schematic diagram of Ti_3_C_2_T_x_ MXene‑modified CF prepared through the molten salt technique; Reproduced with permission [37]. Copyright 2022, American Chemical Society.

Among coating techniques, dip‐coating remains the most straightforward and frequently employed approach. Simple immersion can afford effective binding between Ti_3_C_2_T_x_ MXene and CFs and has enjoyed broad utilization. For example, Lian et al. [44]. immersed CFs in an aqueous Ti_3_C_2_T_x_ MXene solution for 30 min, producing CF composites with excellent electromagnetic wave absorption capabilities: the minimum reflection loss (RL_min_) and effective absorption bandwidth (EAB) reached −57.07 dB and 7.74 GHz, respectively, along with efficient electrothermal conversion and antibacterial properties. Similarly, Zhang et al. [45]. submerged CF/polyether ether ketone (PEEK) composite sheets in a Ti_3_C_2_T_x_ MXene dispersion for 15 min (Figure 2a), yielding CF/PEEK composites with enhanced interfacial properties and EMI shielding performance. The interlaminar shear strength (ILSS) and specific EMI shielding efficiency per unit thickness (EMI SE/t) reached 113.7 MPa and 80.8 dB/mm, respectively. However, a critical challenge of dip‐coating is the stochastic and uncontrolled distribution of Ti_3_C_2_T_x_ MXene on CF surfaces, often resulting in inhomogeneities or locally excessive/deficient coverage, which can compromise the composite's performance.

To improve the precision and uniformity of Ti_3_C_2_T_x_ MXene distribution on CFs, various advanced coating techniques have been developed. For example, Xie et al. [46]. employed spin‐coating, wherein CF fabrics were mounted on a spin coater and uniformly covered with Ti_3_C_2_T_x_ MXene via syringe injection during rotation (Figure 2b), the resulting composite fabrics featured integrated shielding and absorption functions, with an electromagnetic wave absorption rate up to 99.99997% (RL_min_ = ‐65.01 dB), ultralight density (0.279 g/cm^3^), and a record high EAB (10.94 GHz). Shoeb et al. [47]. used drop‐casting to precisely control the mass loading of Ti_3_C_2_T_x_ MXene nanomaterials on CF fabrics (Figure 2c); at an optimal loading of 2.5 mg/cm^2^, the electrodes achieved exceptional areal capacitance of 0.862 F/cm^2^ at a current density of 0.58 mA/cm^2^. Coating techniques such as spin‐coating and drop‐casting offer significant advantages when precise control over Ti_3_C_2_T_x_ MXene‐modified CF is required. However, these methods are generally limited to the preparation of small or even minute quantities of Ti_3_C_2_T_x_ MXene‐modified CF, and their complex procedures are unsuitable for large‐scale production. Therefore, spray‐coating provides a more practical solution for the large‐scale fabrication of Ti_3_C_2_T_x_ MXene‐modified CF. Song et al. [16]. spray‐coated Ti_3_C_2_T_x_ MXene onto CF surfaces and, by controlling the number of sprays passes, achieved uniform coverage and fabricated large‐area EMI shielding composite papers (Figure 2d). A seven‐layer composite exhibited average EMI shielding efficiency (EMI SE) values up to 78.23 dB in the X band. Hu et al. [48]. sprayed a Ti_3_C_2_T_x_ MXene mixture onto the surface of CFs using an electrostatic atomization device, enabling the large‐scale preparation of composites with remarkable EMI shielding effectiveness. When the Ti_3_C_2_T_x_ MXene content ranged from 0.24% to 1.07%, the EMI SE of the composites increased to 87.12 dB, representing an enhancement of 350.02%. Thus, spray‐coating offers a valuable reference for industrial‐scale fabrication of Ti_3_C_2_T_x_ MXene‐modified CFs.

With increased control over precision and scalability, an ongoing challenge remains: how to maximize the adhesion of Ti_3_C_2_T_x_ MXene to CFs while minimizing potential fiber damage from pretreatment. Recent advances indicate that the use of suitable adhesives or coupling agents under mild conditions can significantly improve adhesion. For example, Zou et al. [49]. employed a sequential dip‐coating of polydopamine (PDA) and Ti_3_C_2_T_x_ MXene onto CFs, producing CF/epoxy resin (EP) composites with a hierarchical flexible‐rigid structure. PDA served as a soft interlayer, bridging Ti_3_C_2_T_x_ MXene and CFs through hydrogen bonds and van der Waals forces, significantly enhancing both EMI shielding (68.88 dB) and mechanical properties (tensile modulus and strength reached 542.80 MPa and 7.30 GPa, respectively). Similarly, Chen et al. [50]. improved the mechanical, abrasion, and erosion resistance of CF/EP composites by sequentially dip‐coating CFs with PDA and Ti_3_C_2_T_x_ MXene (Figure 2e): wear rate and erosion volume loss decreased by 79.39% and 66.85%, respectively, compared to unmodified CF/EP composites. PDA can form on the surface of virtually all types of materials under mild conditions. Its abundant active ‐OH groups can further enhance adhesion to Ti_3_C_2_T_x_ MXene through hydrogen bonding interactions, providing a favorable platform for the binding of Ti_3_C_2_T_x_ MXene to CFs [51]. PDA has indeed inspired the preparation of Ti_3_C_2_T_x_ MXene‐modified CFs via coating methods. Similarly, Su et al. [52]. immersed CFs in a Ti_3_C_2_T_x_ MXene solution and the organic binder polyvinyl alcohol (PVA). PVA also enhanced adhesion to Ti_3_C_2_T_x_ MXene through hydrogen bonding; the ILSS of the resulting CF/EP composites increased by 30% compared to unmodified CF/EP composites. Meanwhile, Sun et al. [53]. achieved uniform distribution of Ti_3_C_2_T_x_ MXene on CFs by immersing them in polydimethylsiloxane (PDMS). PDMS provided additional physical sites for the attachment of Ti_3_C_2_T_x_ MXene, forming a more stable interfacial layer. As a result, the interfacial shear strength (IFSS) increased by 113% compared to unmodified CF. Such bridging agents, which can easily form coatings on the CF surface and increase both the number and strength of binding sites between CF and Ti_3_C_2_T_x_ MXene, will further expand the performance potential of Ti_3_C_2_T_x_ MXene‐modified CF.

It is worth noting that some studies have adopted a molten salt process to coat the precursor MAX phase of Ti_3_C_2_T_x_ MXene onto CFs, followed by etching with hydrofluoric acid (HF) to directly obtain CFs with in situ grown Ti_3_C_2_T_x_ MXene. For example, Shi et al. [37]. mixed Ti_2_AlC, aluminum powder, NaCl, and KCl, coated this mixture onto the CF, then melted it in a tube furnace, followed by washing and drying. Subsequently, HF etching yielded Ti_3_C_2_T_x_ MXene‐modified CF for use as capacitor cathodes (Figure 2f). The resulting capacitors displayed a high areal capacitance of 380 mF/cm^2^ and excellent cycle stability, maintaining 90% capacitance retention after 10,000 cycles. Similarly, Zhang et al. [54]. used the same method to prepare Ti_3_C_2_T_x_ MXene‐modified CFs as free‐standing battery anodes. After 400 cycles at a current density of 1 mA cm^−2^, the lithium‐ion charge/discharge capacity reached 269 mAh g^−1^; and after 100 cycles at 0.1 mA cm^−2^, the sodium‐ion charge/discharge capacity was 206 mAh g^−1^. This innovative design strategy not only saves the complicated step of collecting Ti_3_C_2_T_x_ MXene nanosheets from solution but also reduces the adverse effects caused by the re‐stacking of Ti_3_C_2_T_x_ MXene nanosheets on CF, which can otherwise complicate ion transport channels. Therefore, it offers unique advantages in applications such as battery energy storage.

In summary, the coating method demonstrates strong adaptability and lasting vitality in the field of Ti_3_C_2_T_x_ MXene‐modified CF. With currently mature coating techniques, it possesses considerable design flexibility and practical feasibility. However, certain limitations remain. At present, the coating method is mostly suitable for the treatment of quantified CF batches, and the relatively long processing times create a significant gap between this approach and the requirements of industrial‐scale production of Ti_3_C_2_T_x_ MXene‐modified CF. It should be especially emphasized that, in terms of modification mechanism, the adhesion between Ti_3_C_2_T_x_ MXene and CF achieved by coating is primarily dominated by weak secondary interactions (such as hydrogen bonds and van der Waals forces). Therefore, the insufficient bonding strength of Ti_3_C_2_T_x_ MXene on CF prepared by coating remains a key issue that requires particular attention. Future research should focus on: developing more efficient and stable coating systems to enhance both the adhesion and durability of Ti_3_C_2_T_x_ MXene; optimizing coating and spraying parameters to better integrate nanoscale uniformity with industrial‐scale throughput; deepening the mechanistic understanding and controllability of novel methods such as molten salt in situ growth; and exploring synergistic strategies that combine coating with other modification techniques, in order to fully unlock the multidimensional application potential of Ti_3_C_2_T_x_ MXene‐modified CFs in industrial production.

### 2.2 Self‐Assembly

Self‐assembly is a molecular organization technique based on electrostatic interactions, whereby oppositely charged species (such as polyelectrolytes, nanoparticles, or biomolecules) spontaneously form ordered multilayer structures at solid‐liquid interfaces or in solution through attractive forces [55, 56]. This method is not constrained by the shape or size of the substrate and enables the tailored, hierarchical structuring of CF surfaces [57]. By introducing oppositely charged species onto the CF surface, hierarchical self‐assembly can be achieved through electrostatic interactions, garnering considerable interest in the field of CF modification. Notably, the abundance of negatively charged surface groups on Ti_3_C_2_T_x_ MXene makes it ideally suited for such self‐assembly with positively charged modifiers [58]. Currently, the prevailing approach for preparing Ti_3_C_2_T_x_ MXene‐modified CFs via self‐assembly involves first grafting cationic species onto the CFs, followed by the electrostatic attraction of Ti_3_C_2_T_x_ MXene onto the fiber surface [59]. Numerous studies have demonstrated that electrostatic interactions yield significantly stronger adhesion compared to hydrogen bonding or van der Waals forces, suggesting that self‐assembly results in enhanced binding strength between Ti_3_C_2_T_x_ MXene and CFs, and offers substantial application potential [60].

**FIGURE 3:**
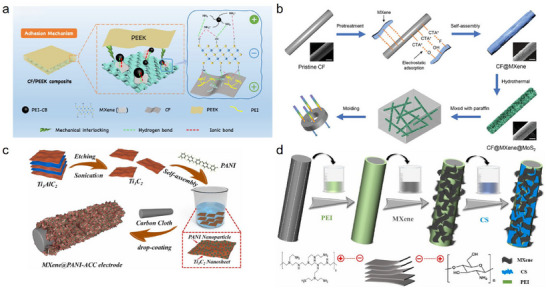
(a) Schematic diagram of Ti_3_C_2_T_x_ MXene‑modified CF prepared through PEI‐driven self‑assembly process; Reproduced with permission [61]. Copyright 2025, Elsevier. (b) Schematic diagram of Ti_3_C_2_T_x_ MXene‑modified CF prepared through PEI‐driven self‑assembly process; Reproduced with permission [63]. Copyright 2020, Wiley. (c) Schematic diagram of Ti_3_C_2_T_x_ MXene‑modified CF prepared through PANI‐driven self‑assembly process; Reproduced with permission [66]. Copyright 2022, Elsevier. (d) Schematic diagram of the chitosan‑assisted self‑assembly fabrication of Ti_3_C_2_T_x_ MXene‑modified CF; Reproduced with permission [69]. Copyright 2021, Elsevier.

To realize deposition of Ti_3_C_2_T_x_ MXene onto CFs via self‐assembly, grafting suitable positively charged species onto the CF surface is essential. At present, the mainstream choice is cationic polyelectrolytes that possess good solubility, excellent film‐forming ability, and can ionize to release sufficient cations. Polyamines, such as polyetherimide (PEI), are exemplary cationic polyelectrolytes; their abundant amine groups confer considerable net positive charge (‐NH_3_^+^, ‐NRH_2_^+^, ‐NR_2_H^+^) in aqueous solution, facilitating electrostatic attachment to both the CF surface and subsequent Ti_3_C_2_T_x_ MXene deposition [56]. For example, Zhou et al. [61]. treated CFs in a 1 mg/mL PEI aqueous solution for 1 h before immersing them in a Ti_3_C_2_T_x_ MXene suspension for 30 min to complete the self‐assembly process (Figure 3a). The resulting CF/PEEK composites exhibited outstanding mechanical and electromagnetic shielding performance, compared to unmodified composites, ILSS increased by 60.27% and EMI SE by 33.56%. Xu et al. [62]. employed self‐assembly between PEI and Ti_3_C_2_T_x_ MXene/CNTs nanohybrids, creating modified CFs with a 1D/2D hybrid surface topology, leading to enhancements in flexural strength, modulus, and ILSS by 74.16%, 63.76%, and 62.79%, respectively.

Another common class of cationic polyelectrolytes is polyquaternary ammonium salts such as cetyltrimethylammonium bromide (CTAB) (Figure 3b). This type of modifier features a stable structure and strong cationic character, thereby efficiently facilitating the electrostatic self‐assembly of Ti_3_C_2_T_x_ MXene [63]. For instance, Han et al. [64]. used CTAB‐Ti_3_C_2_T_x_ MXene self‐assembly combined with carboxymethyl cellulose (CMC) to prepare CFs with a flexible‐rigid structure, achieving a 102.7% increase in IFSS of CF/EP composites compared to unmodified ones. Wang et al. [65]. similarly developed novel electrochemical sensors were developed by self‐assembling CTAB with Ti_3_C_2_T_x_ MXene on CFs, realizing high sensitivity, selectivity, and stability for kaempferol (KA) detection.

Conductive polymers such as polyaniline (PANI) have also been introduced into self‐assembly processes with Ti_3_C_2_T_x_ MXene (Figure 3c), offering high conductivity, chemical stability, and the added benefit of suppressing Ti_3_C_2_T_x_ MXene restacking [66]. Ma et al. [67]. formed modified CF electrodes via self‐assembly of PANI and Ti_3_C_2_T_x_ MXene, achieving asymmetric supercapacitors with exceptionally wide voltage windows (1.1 V) and high specific capacitance (400 F/g at 1 A/g), owing to the combined electronic and ionic transport characteristics. Yin et al. [68]. constructed flexible composites with high EMI shielding by coassembling PANI and Ti_3_C_2_T_x_ MXene onto CFs, yielding an EMI SE of 35.3 dB at a thickness of only 0.376 mm. The diversity of cationic donors available for CF modification via self‐assembly not only expands the strategy's versatility but also enhances the potential for further functional applications of Ti_3_C_2_T_x_ MXene‐modified CFs.

With the application of increasingly novel self‐assembly strategies in Ti_3_C_2_T_x_ MXene‐modified CFs, more types of modification materials have also been employed. These modifiers not only address the stacking and agglomeration of Ti_3_C_2_T_x_ MXene nanoparticles and stabilize the hierarchical structure of Ti_3_C_2_T_x_ MXene‐modified CF, but also synergistically participate in the self‐assembly process of Ti_3_C_2_T_x_ MXene. As a result, they can significantly enhance the potential of Ti_3_C_2_T_x_ MXene in various application fields. These co‐modifiers, which include chitosan (Figure 3d) [69], conductive carbon black [61], CNT [62], RuO_2_ [65], graphene oxide [70], SiO_2_ [71], and so on, participate synergistically in the self‐assembly process, and their incorporation in different dimensions and morphologies enables the rational, multiscale engineering of hierarchical CF surfaces, further boosting CFs and CFRPs performance.

Overall, electrostatic self‐assembly offers a highly attractive route for the fabrication of Ti_3_C_2_T_x_ MXene‐modified CFs. Its major advantages include substrate universality (regardless of shape and size), operational simplicity, exceptionally robust interfacial adhesion (far exceeding that attained by hydrogen bonding or van der Waals forces), and tremendous scalability and structural design freedom. However, challenges remain: long processing times limit its suitability for large‐scale industrial production, and the stability of deposited Ti_3_C_2_T_x_ MXene on CFs may be inadequate under practical conditions. Future research should focus on developing more efficient cationic modification methodologies, refining precision in constructing multidimensional heterostructures, deepening the mechanistic understanding and process control, and facilitating the transition of this technique to scalable, industrial implementations—thus fully unlocking the tremendous potential of Ti_3_C_2_T_x_ MXene‐modified CFs across major technological domains.

### 2.3 Electrophoretic Deposition (EPD)

In the past decade, EPD has emerged as an effective technology for the fabrication of nanomaterial‐modified CFs, becoming a research hotspot in CF modification. The fundamental principle of EPD is that, under a direct current electric field, charged nanoparticles in a suspension migrate toward CF electrodes bearing opposite charges as a result of Coulombic forces, upon contacting the CF electrode surface, the particles’ charges are neutralized, resulting in their deposition on the fiber [9, 29]. This process offers the dual benefits of high production efficiency and superior deposition quality. Particles can be deposited on CFs in a short period, and the deposited amount can be precisely controlled by adjusting process parameters such as the deposition voltage and time. EPD also enables uniform nanoparticle deposition on CFs at low solution concentrations, which is highly advantageous for large‐scale, continuous industrial production [72]. Ti_3_C_2_T_x_ MXene possesses inherent advantages for EPD‐based CF modification owing to its high electrical conductivity and abundant negative surface charge, facilitating efficient deposition onto CFs without the need for additional binders. The EPD process also effectively preserves the original morphology and functional groups of Ti_3_C_2_T_x_ MXene, minimizing inherent defects caused by external forces and protecting the layer‐to‐layer connectivity, this provides a solid foundation for Ti_3_C_2_T_x_ MXene to exploit its structural advantages on CFs [73]. Since its first successful application to Ti_3_C_2_T_x_ MXene‐modified CFs in 2022, EPD has become an ideal and cost‐effective choice for many researchers [27].

**FIGURE 4:**
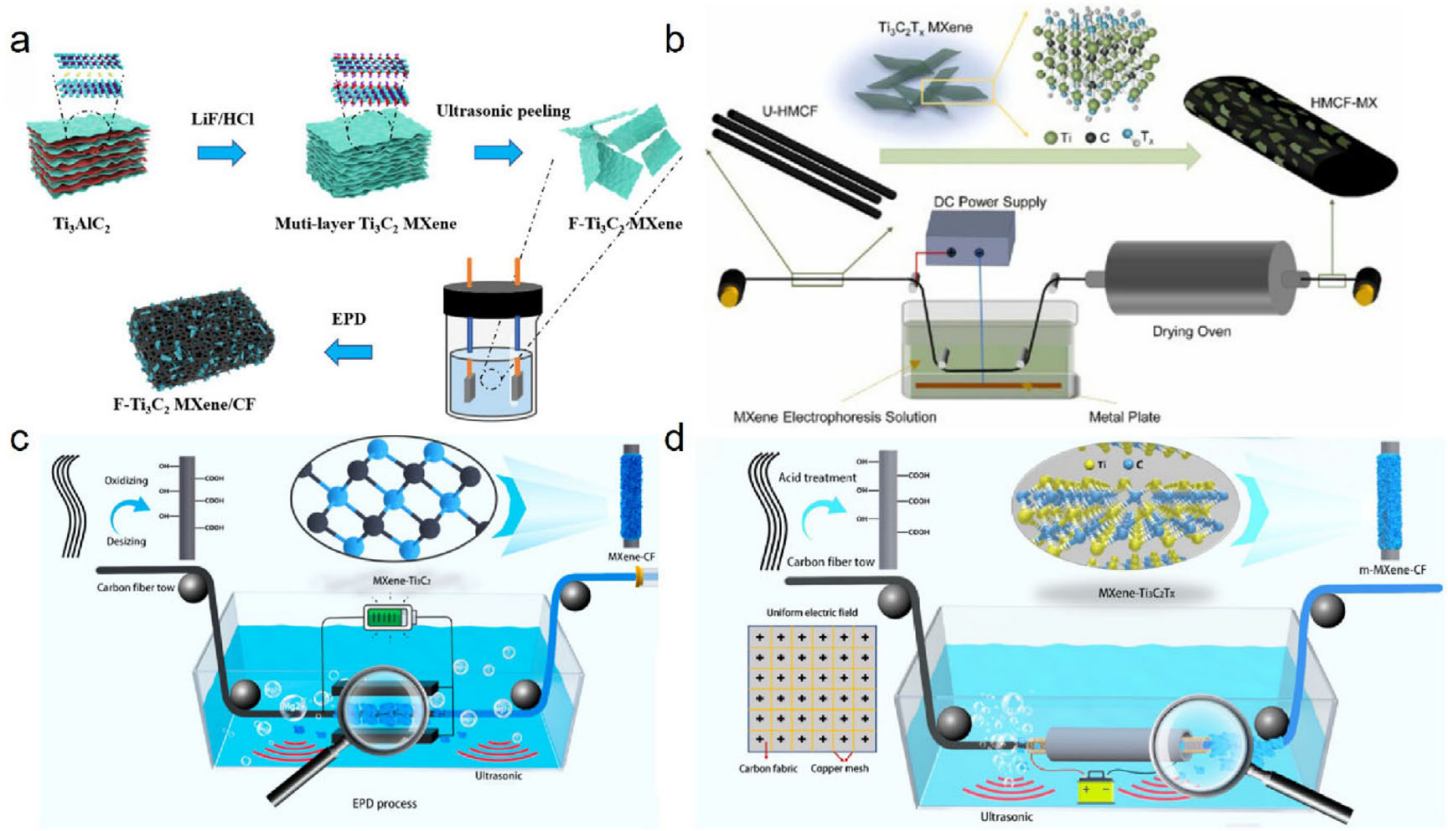
(a) Schematic diagram of Ti_3_C_2_T_x_ MXene‑modified CF electrode prepared through EPD method; Reproduced with permission [74]. Copyright 2024, Elsevier. (b) Schematic diagram of Ti_3_C_2_T_x_ MXene‑modified CF prepared through anodic deposition process; Reproduced with permission [75]. Copyright 2023, Elsevier. (c) Schematic diagram of Ti_3_C_2_T_x_ MXene‑modified CF prepared through cathodic deposition process; Reproduced under terms of the CC‐BY license [76]. Copyright 2023, Elsevier. (d) Schematic diagram of Ti_3_C_2_T_x_ MXene‑modified CF prepared through EPD process with ultrasonic assistance and optimized electric‑field distribution; Reproduced with permission [77]. Copyright 2022, Elsevier.

Thanks to its high negative surface charge, Ti_3_C_2_T_x_ MXene migrates toward positive electrodes in solution, making anodic deposition a logical approach for preparing Ti_3_C_2_T_x_ MXene‐modified CFs. For example, Wang et al. [74]. utilized anodic deposition at 30 V for 30 s, achieving direct fabrication of Ti_3_C_2_T_x_ MXene‐modified CF electrodes without any binder (Figure 4a). The resulting electrodes exhibited a remarkable increase in the number of surface functional groups and enhanced electrochemical activity, inactivating Escherichia coli with an efficiency as high as 99.99% within 60 min. Huang et al. [75]. developed a continuous anodic deposition process to prepare Ti_3_C_2_T_x_ MXene‐modified high modulus CFs (HMCFs) with a deposition voltage of 15 V and a duration of 120 s (Figure 4b). The IFSS and ILSS of the HMCF/EP composites reached 93.4 MPa and 86.8 MPa, representing 46.9% and 49.9% improvements over the unmodified composites, respectively.

Cathodic deposition is also feasible, achieved by reversing the electrical behavior of Ti_3_C_2_T_x_ MXene, often via co‐deposition with metal ions. The underlying mechanism involves charge reversal: positively charged metal ions (e.g., Mg^2+^) adsorb onto the negatively charged surface of Ti_3_C_2_T_x_ MXene nanosheets via electrostatic interactions or coordination, neutralizing or even reversing their net surface charge [29]. As a result, under an electric field, the modified MXene migrates toward the cathode. For instance, Hu et al. [76]. introduced hexahydrate magnesium chloride into the Ti_3_C_2_T_x_ MXene suspension to alter its electronegativity and then used cathodic deposition to deposit Ti_3_C_2_T_x_ MXene nanoparticles on CFs (Figure 4c). The resulting CFRP exhibited significantly enhanced mechanical and electrical properties, compared to unmodified CFRP, flexural strength and through‐thickness conductivity improved by 26% and 66%, respectively. Hu et al. [40]. further introduced Ti_3_C_2_T_x_ MXene onto electroplated nickel (Ni)‐coated CFs via a similar cathodic deposition method, resulting in composites with outstanding EMI SE up to 72.4 dB. Reports indicate that the use of charge reversal agents in cathodic deposition can further strengthen the migration drive and increase the deposition rate [29]. As research into EPD‐based fabrication of Ti_3_C_2_T_x_ MXene‐modified CFs progresses, refining and optimizing the EPD process remains a crucial area for ongoing investigation. At present, more uniform deposition of Ti_3_C_2_T_x_ MXene on CFs has been effectively achieved through methods such as ultrasonic assistance [27] and optimization of the electric field distribution (Figure 4d) [77]. These approaches help to fully exploit both the process advantages of EPD and the performance advantages of Ti_3_C_2_T_x_ MXene‐modified CF.

Overall, EPD is now regarded as a “star” technique for Ti_3_C_2_T_x_ MXene‐CF fabrication due to its efficiency, controllability, and scalability. This approach utilizes the electric field‐driven directional migration and deposition of Ti_3_C_2_T_x_ MXene nanosheets, achieving uniform, high‐efficiency loading onto CF surfaces without requiring external adhesives. Importantly, EPD maintains the intrinsic structure, functional activity, and layered connectivity of MXene, providing a solid foundation for maximizing its electrical conductivity and surface activity advantages. Nevertheless, the nature of the bonding between Ti_3_C_2_T_x_ MXene and CFs during EPD still requires further clarification, and challenges such as insufficient bonding strength may persist. Future research directions should include deepening the understanding of how EPD process parameters (such as voltage, time, and suspension properties) influence interfacial structure and performance; optimizing equipment for continuous EPD processing of CFs; and exploring the synergistic effects of EPD‐fabricated Ti_3_C_2_T_x_ MXene/CF materials in multifunctional composites. These efforts will be critical to further advancing the industrial application and sustained development of EPD‐based Ti_3_C_2_T_x_ MXene‐modified CFs.

### 2.4 Chemical Grafting

**FIGURE 5:**
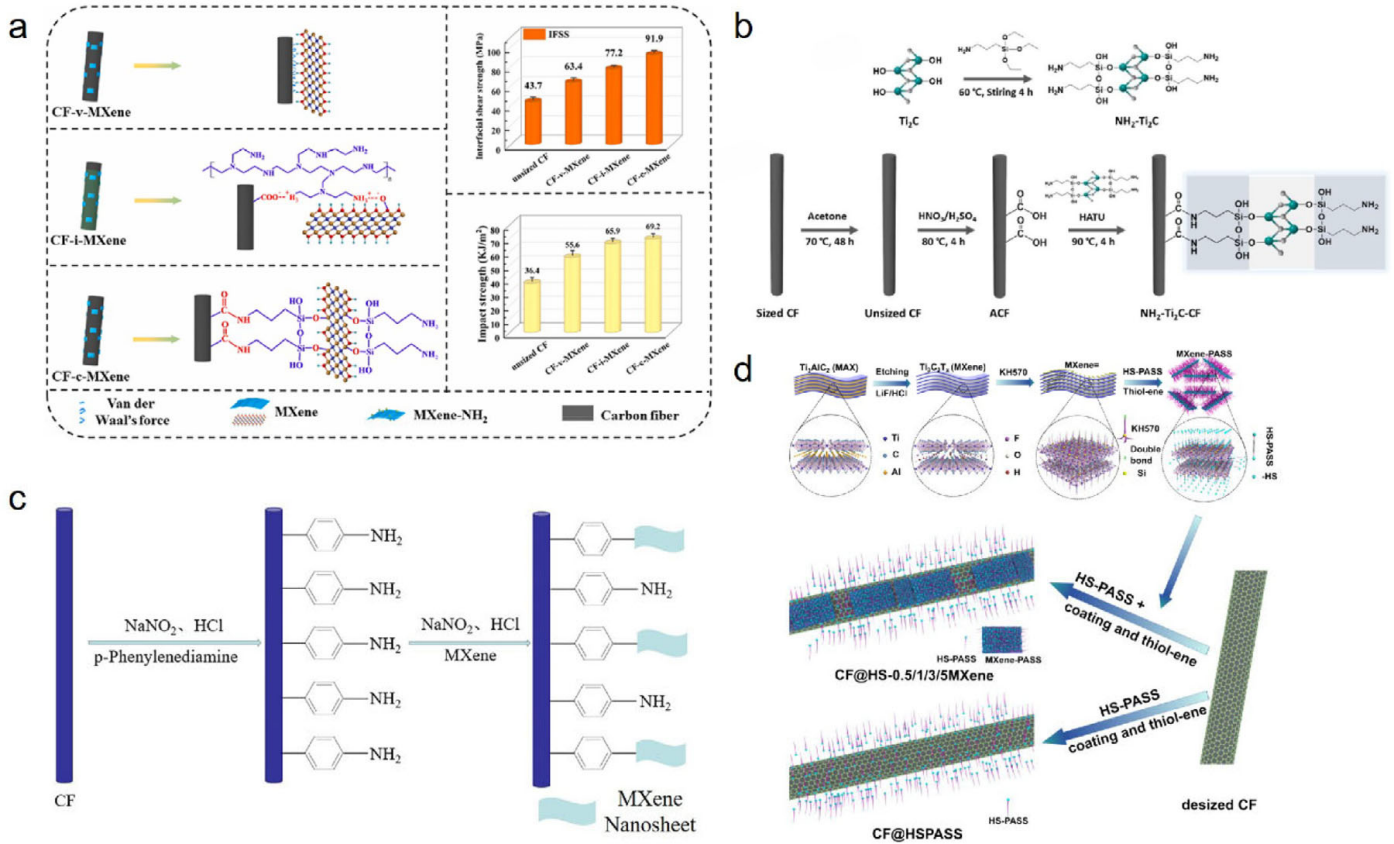
(a) Comparison of various bonding types and interfacial performance of Ti_3_C_2_T_x_ MXene‑modified CF composites; Reproduced with permission [80]. Copyright 2023, Elsevier. (b) Schematic diagram of covalent bonding between Ti_3_C_2_T_x_ MXene‑modified CFs via ‐NH_2_ groups; Reproduced with permission [13]. Copyright 2020, Elsevier. (c) Schematic diagram of preparing Ti_3_C_2_T_x_ MXene‑modified CF by electrochemistry aryl diazonium salt reaction; Reproduced with permission [87]. Copyright 2022, Elsevier. (d) Schematic diagram of Ti_3_C_2_T_x_ MXene‑modified CF prepared via thiol‑ene click reaction; Reproduced with permission [88]. Copyright 2025, American Chemical Society.

Chemical grafting is a modification technique that introduces reactive functional groups onto the nonpolar surface of CFs via chemical reactions, thus enabling the formation of covalent bonds between the modifier and the CF [78]. This approach is equally applicable to the integration of Ti_3_C_2_T_x_ MXene with CFs, as the abundant surface functional groups and reactive sites on Ti_3_C_2_T_x_ MXene provide expansive possibilities for chemical grafting, making it a research hotspot in recent years for the development of Ti_3_C_2_T_x_ MXene‐modified CFs. Crucially, studies have demonstrated that the introduction of covalent bonding significantly enhances the performance of Ti_3_C_2_T_x_ MXene‐modified CFs. For example, Ma et al. [30]. compared the effects of hydrogen bonding, van der Waals forces, and covalent interactions on the IFSS and ILSS of Ti_3_C_2_T_x_ MXene‐modified CF/EP composites. The results showed that covalent bonds were most effective, producing the highest IFSS (56.39 MPa) and ILSS (60.72 MPa), a benefit attributed to the exceptional stability of covalently tethered Ti_3_C_2_T_x_ MXene. Zhuang et al. [79]. further assessed the hydrothermal aging behavior of such composites and confirmed that covalent bonding conferred superior reliability, maintaining the highest ILSS (50 MPa) even after prolonged hydrothermal treatment. In another comprehensive study, Hu et al. [80]. incorporated electrostatic interactions into their comparison and concluded that covalent attachment endowed Ti_3_C_2_T_x_ MXene‐modified CFs with the greatest surface roughness, number of active sites, and interfacial adhesion, ultimately enhancing the composite's mechanical properties (Figure 5a). Thus, chemical grafting provides strong interfacial bonding between Ti_3_C_2_T_x_ MXene and CFs through the formation of covalent bonds, which is far superior to the electrostatic interactions, van der Waals forces, and hydrogen bonds offered by other methods. This approach maximizes the interfacial adhesion enhancement effect of Ti_3_C_2_T_x_ MXene, offering a novel strategy for preparing Ti_3_C_2_T_x_ MXene‐modified CF with outstanding performance advantages.

A central challenge in chemical grafting lies in the introduction of suitable reactive groups on both CF and Ti_3_C_2_T_x_ MXene surfaces. The predominant strategy is to functionalize Ti_3_C_2_T_x_ MXene with amino (‐NH_2_) groups, enabling the formation of covalent bonds with ‐COOH or other functional groups on pretreated CFs [81]. For instance, Ding et al. [13]. utilized the silanol hydroxyl groups (Si‐OH) from hydrolyzed 3‐aminopropyltriethoxysilane (APTES) to condense with the active ‐OH groups on Ti_3_C_2_T_x_ MXene, thereby achieving amino‐functionalization of Ti_3_C_2_T_x_ MXene (Figure 5b). Subsequently, the ‐NH_2_ groups on the MXene reacted with ‐COOH groups on CF to form amide bonds (‐NHCO‐), leading to the preparation of Ti_3_C_2_T_x_ MXene‐modified CF. The resulting CF/EP composites exhibited IFSS and ILSS values of 72.17 and 44.24 MPa, respectively. These values represent increases of 77.9% and 27.8% compared to unmodified CF/EP composites, indicating a significant reinforcement effect. Quan et al. [82]. achieved chemical grafting between CF and Ti_3_C_2_T_x_ MXene by utilizing APTES‐modified Ti_3_C_2_T_x_ MXene and phytic acid (PA)‐treated CF, this process mainly relied on the abundant phosphate groups in PA forming covalent bonds (‐C/P═O─NH‐) with the ‐NH_2_ groups of APTES. Compared to untreated CFRP, the modified CFRP showed improvements of 51.4% in flexural strength, 62.5% in flexural modulus, 63.0% in ILSS, 74.9% in tensile strength, and 45.8% in IFSS. Moreover, owing to the excellent reactivity between the silane coupling agent APTES, Ti_3_C_2_T_x_ MXene, and CF, researchers are no longer limited to APTES as the only silane agent providing ‐NH_2_ groups. Han et al. [83]. demonstrated that, besides APTES, 3‐glycidyloxypropyl‐dimethoxymethylsilane (GPDMS) and 3‐mercaptopropyltrimethoxysilane (MPTS) could also modify MXene, forming stable Si─O─C bonds with CF ‐OH groups, thereby significantly enhancing interfacial adhesion. These findings greatly expand the versatility of silane coupling agents in the chemical grafting of Ti_3_C_2_T_x_ MXene on CFs and open further avenues for interface engineering.

However, the aforementioned designs typically require multiple sequential treatments of both Ti_3_C_2_T_x_ MXene and CF, leading to extended processing times and limited efficiency. To address this, electrochemical aryl diazonium grafting has emerged as a new strategy. Here, aromatic amines react with nitrite or strong acids to produce aryl diazonium salts, which are subsequently reduced in acidic electrolytes to generate radicals that covalently anchor to the substrate [84]. The diazonium approach is prized for its efficiency, controllability, and substrate compatibility, without causing damage to the base material [85]. For instance, Coia et al. [86]. employed this reaction to covalently tether anthraquinone derivatives onto CF surfaces, which then coordinated with Ti_3_C_2_T_x_ MXene via C═O groups, substantially increasing the capacitance of the resulting Ti_3_C_2_T_x_ MXene‐modified CF electrodes (up to 31.7 F/g) while maintaining the fiber's mechanical integrity. Chen et al. [87]. implemented a similar method using p‐phenylenediamine as a bridging molecule, achieving Ti‐O‐C covalent bonds between Ti_3_C_2_T_x_ MXene and CF (Figure 5c). The IFSS of the resulting composite reached 123.86 MPa, 33.5% higher than the unmodified control, with no decrease in tensile strength. Such highly efficient, non‐destructive methods present exciting new directions and great potential for fabricating Ti_3_C_2_T_x_ MXene‐modified CFs.

Recently, innovative chemical grafting concepts have also emerged. Zhang et al. [88]. functionalized Ti_3_C_2_T_x_ MXene with C═C bonds using 3‐(trimethoxysilyl)propyl methacrylate, then achieved covalent linkage with PASS resin‐modified CFs via thiol‐ene “click” chemistry (Figure 5d). The resulting CFRPs exhibited synergistic enhancement in both mechanical and EMI shielding performance, with IFSS and ILSS increased by 59.2% and 109.1%, respectively, and EMI SE reaching 22 dB in the 8.2–12.4 GHz range compared to unmodified CFRP. It is anticipated that, in the future, a wider variety of efficient and suitable chemical grafting strategies will be applied to the preparation of Ti_3_C_2_T_x_ MXene‐modified CFs, injecting new vitality and even greater possibilities into the fabrication of high‐performance CFs.

In summary, chemical grafting creates robust covalent interfaces between Ti_3_C_2_T_x_ MXene and CF, providing a powerful strategy for high‐performance CFRP design. Covalent bonding, with its unrivaled interfacial strength compared to hydrogen bonding, van der Waals, or electrostatic interactions, underpins the property breakthroughs of Ti_3_C_2_T_x_ MXene‐modified CFs. Current research on chemical grafting has deepened our understanding of interfacial engineering in Ti_3_C_2_T_x_ MXene‐modified CFs and is driving paradigm shifts in the fabrication of advanced, multifunctional composites. However, challenges remain: chemical grafting typically involves lengthy processes that limit scalability and efficiency, and often requires large quantities of solvent, raising environmental and recovery concerns. In the future, it will be important to develop efficient and universal grafting techniques, as well as to advance one‐step or in situ grafting strategies to simplify processing procedures and reduce energy consumption, thereby promoting large‐scale applications. In addition, exploring molecular structure regulation of grafting agents (such as introducing flexible segments or functional groups) is essential for simultaneously optimizing the mechanical, electrical, and environmental response properties of composites. Furthermore, the development of green and sustainable grafting processes (such as aqueous systems or solvent‐free grafting technologies) will help reduce the use of toxic reagents, aligning with the trend toward environmentally friendly manufacturing.

### 2.5 Blending‐Spinning and Carbonization

Recent advances in the study of Ti_3_C_2_T_x_ MXene‐modified CFs have highlighted the integration of outstanding mechanical, electrical, thermal, and chemical properties as a central aim. Nevertheless, conventional preparation approaches typically confine Ti_3_C_2_T_x_ MXene to the CF surface, raising concerns regarding interfacial adhesion and significant loss of material efficiency due to surface stacking of Ti_3_C_2_T_x_ MXene nanosheets [89]. A promising solution is to hybridize Ti_3_C_2_T_x_ MXene within the fiber interior, thereby enhancing the degree and uniformity of Ti_3_C_2_T_x_ MXene loading. This prospect has led to the adoption of blending‐spinning technology. In this approach, Ti_3_C_2_T_x_ MXene nanosheets are mixed with the CF precursor and subsequently processed via wet spinning or electrospinning, enabling precise control over fiber structure and the integration of multifunctionality, subsequent annealing and carbonization of these hybrid fibers yield Ti_3_C_2_T_x_ MXene‐modified CFs [90, 91]. This method not only improves MXene loadings and exposes more active sites, but also imparts exceptional physicochemical attributes (such as high specific surface area, enhanced electrical conductivity, and optimal porosity) that promise broad application prospects in high‐precision domains [92, 93].

**FIGURE 6:**
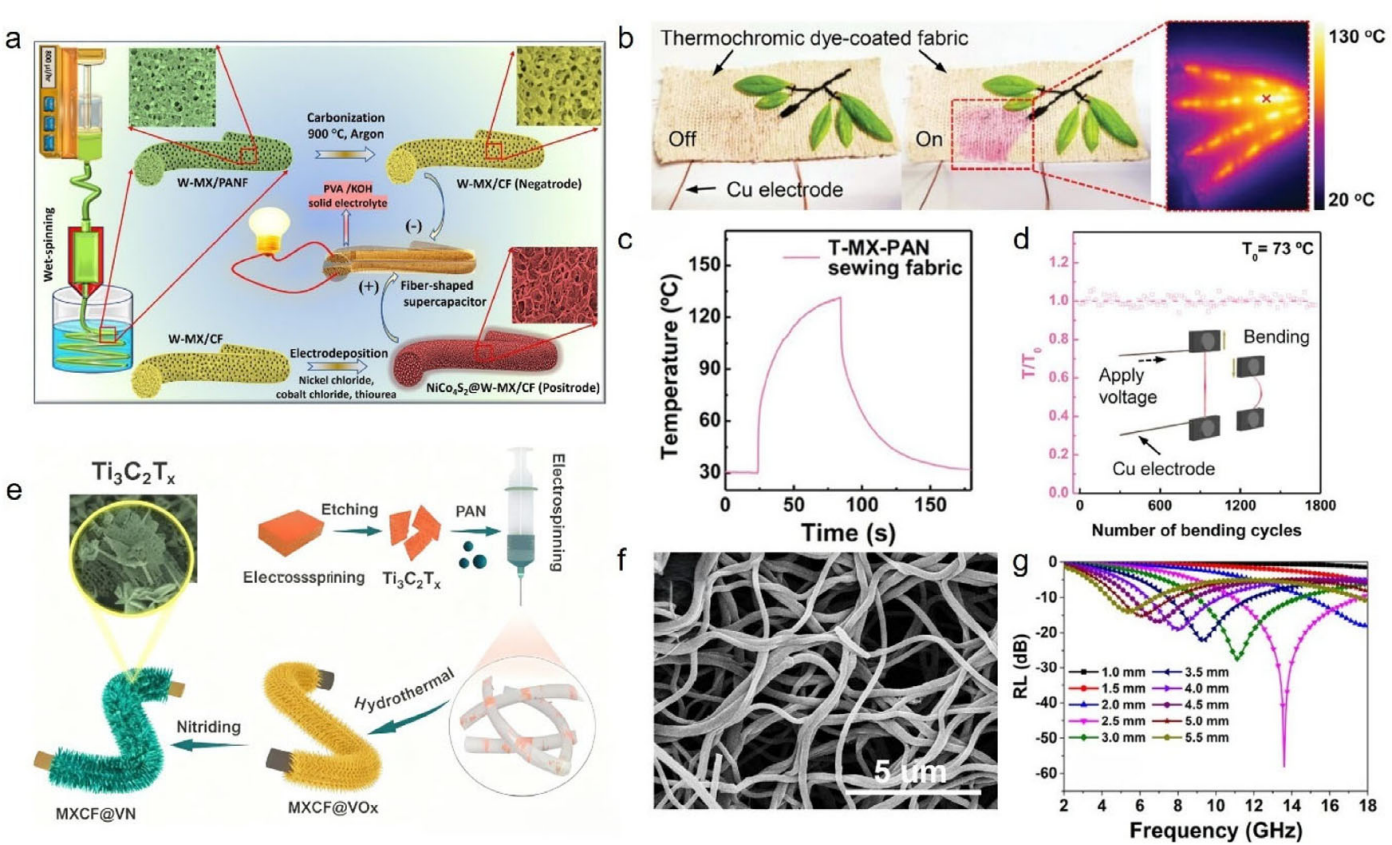
(a) Schematic diagram of wet‑spinning fabrication of porous Ti_3_C_2_T_x_ MXene‑modified CF for developing a solid‑state asymmetric supercapacitor; Reproduced with permission [95]. Copyright 2022, Elsevier. (b) Photographs and IR images of Ti_3_C_2_T_x_ MXene‑modified CFs sewn onto thermochromic dye‐coated fabrics before and after the application of voltage, and (c) Corresponding time‐temperature curve. (d) Retention of temperature performance of Ti_3_C_2_T_x_ MXene‑modified CF after 1800 bending cycles. (b–d) Reproduced with permission [96]. Copyright 2024, Wiley. (e) Schematic diagram of electrospinning fabrication of the porous Ti_3_C_2_T_x_ MXene‑modified CF electrode; Reproduced with permission [97]. Copyright 2024, Royal Society of Chemistry. (f) SEM image of Ti_3_C_2_T_x_ MXene‑modified CFs fabricated by electrospinning and carbonization. (g) The EMW absorption performances of Ti_3_C_2_T_x_ MXene‑modified CF. (f, g) Reproduced with permission [98]. Copyright 2021, American Chemical Society.

Wet spinning has long been a core technology in the production of polyacrylonitrile (PAN)‐based CFs. Typically, PAN solutions are extruded through spinnerets into a coagulation bath (e.g., water or an organic solvent), where double‐diffusion effects (solvent diffusion out, non‐solvent diffusion in) induce fiber solidification. This technique also enables effective integration of Ti_3_C_2_T_x_ MXene into CFs [2, 94]. For instance, Khumujam et al. [95]. combined wet spinning of PAN/Ti_3_C_2_T_x_ MXene blends with subsequent carbonization under argon to produce porous, free‐standing CF electrodes (Figure 6a). This exhibited high porosity and a specific surface area up to 550.25 m^2^/g. At a Ti_3_C_2_T_x_ MXene‐to‐PAN mass ratio of 4:5, the resultant CF electrodes achieved a high specific capacitance of 2160 mF/cm^2^ at a current density of 3.86 mA/cm^2^, far surpassing the 680 mF/cm^2^ obtained from pure PAN‐derived CFs. Similarly, Jeong et al. [96]. blended equal volumes of Ti_3_C_2_T_x_ MXene and PAN, followed by wet spinning and carbonization, to manufacture Ti_3_C_2_T_x_ MXene‐modified CFs with robust electrical and thermal performance even under harsh conditions (Figure 6b,c). Notably, these fibers retained flexibility through 1800 bending cycles, evidencing comprehensive multifunctional enhancement (Figure 6d).

Electrospinning, a relatively simple and cost‐effective method for nanofiber fabrication, utilizes a high‐voltage electric field to generate charged Taylor cones from a polymer solution or melt. As the resulting nanoscale jets are stretched and the solvent evaporates (or the melt solidifies), ultrafine fibers are deposited. This approach is also suitable for spinning PAN/Ti_3_C_2_T_x_ MXene blends [93]. For example, Zhang et al. [97]. loaded a mixed solution of PAN and Ti_3_C_2_T_x_ MXene into a syringe, and, by applying a voltage of 20 kV, used electrospinning followed by pre‐oxidation and carbonization to produce porous, Ti_3_C_2_T_x_ MXene‐modified CF electrode materials (Figure 6e). The as‐prepared fibers displayed enhanced specific surface area and abundant active sites, resulting in flexible devices with outstanding energy density (83.95 Wh·kg^−1^) and robust cycling stability, maintaining 82.8% capacity retention after 20,000 cycles. Wang et al. [98]. prepared Ti_3_C_2_T_x_ MXene‐modified CF by employing electrospinning and carbonization techniques, where a mixture of PAN and Ti_3_C_2_T_x_ MXene was processed under a high voltage of 12 kV and carbonized at 650°C in a nitrogen atmosphere. The SEM image of the resulting CF is shown in Figure 6f. The resulting CF composite fabric exhibited excellent electromagnetic wave absorption properties, achieving an RL of −58.0 dB and an EAB of 7.0 GHz at a thickness of 2.5 mm (Figure 6g). The integration of blending‐spinning and carbonization has thus opened novel avenues for Ti_3_C_2_T_x_ MXene‐modified CF production and inspired new strategies for nanoscale fiber modification.

In summary, blending‐spinning techniques (including wet spinning and electrospinning) enable molecular‐level integration of Ti_3_C_2_T_x_ MXene nanosheets with PAN precursors, achieving homogeneous internal loading of Ti_3_C_2_T_x_ MXene within CFs. This approach overcomes the limitations of conventional surface‐only modifications, such as stacking effects and insufficient interfacial adhesion. Nevertheless, several key challenges remain: high‐temperature carbonization may damage Ti_3_C_2_T_x_ MXene structure, leading to loss of electrical conductivity and active sites; the low throughput of electrospinning and the high solvent recovery cost of wet spinning restrict scalability. In the future, the development of this process can focus on several directions: developing low‐temperature catalytic carbonization or plasma‐assisted carbonization technologies to better preserve the intrinsic structure of Ti_3_C_2_T_x_ MXene; optimizing spinning parameters (such as coagulation bath composition and electric field strength) to establish more universal and scalable spinning procedures; introducing multifunctional fillers to enhance the integrated multifunctionality of the fibers; conducting systematic studies on the compatibility and dispersion stability of Ti_3_C_2_T_x_ MXene nanosheets with PAN molecular chains as well as the structural evolution during carbonization, to reinforce the mechanical properties and long‐term reliability of the fibers; and developing green solvent‐based spinning systems (such as ionic liquid‐ or water‐based coagulation baths) to reduce production energy consumption and environmental pollution.

### 2.6 Comparison of Different Preparation Techniques and Selection Strategies

Various preparation techniques (such as coating, self‐assembly, EPD, chemical grafting, blending‐spinning and carbonization) provide a rich toolbox for the development of Ti_3_C_2_T_x_ MXene‐modified CFs. However, these methods differ significantly in terms of their principles, advantages and disadvantages, and application orientations. To clarify the distinct strengths of each technique and offer guidance for material design tailored to specific performance goals, this section conducts a systematic horizontal comparison and summarizes selection strategies (Table 1).

**TABLE 1:** Comprehensive Comparison of Major Preparation Techniques for Ti_3_C_2_T_x_ MXene‐Modified CFs.

Technical Category	Core Principle	Major Advantages	Major Limitations	Typical Application	Refs.
Coating Techniques	Physical adsorption/Solution wetting	Simple, low cost, scalable, widely applicability	Weak bonding, non‐uniform distribution	EMI shielding, surface conductive layer	[44, 46]
Self‐Assembly	Layer‐by‐Layer (LbL) assembly via electrostatic/molecular interactions	Uniform controllable layers, strong interface, multi‐dimensional structures	Time‐consuming, sensitive to solution environment, multilayers may affect fiber flexibility	Flexible electrodes, multilayer functional devices	[67, 68]
EPD	Electric field‐driven deposition of charged particles	Efficient, rapid, uniform, binder‐free, continuous process compatible	Complex parameter optimization, bonding mechanism unclear	Continuous CFRP production, integrated sensors	[75, 76]
Chemical Grafting	Covalent bonding via surface functional groups	Exceptional bonding strength, high durability	Time‐consuming, harmful reagents, fiber damage risk	Extreme‐environment CFRP, long‐life electrodes, high‐reliability sensors	[85, 86]
Blending‐Spinning and Carbonization	Molecular‐level blending of MXene with precursor & carbonization	Uniform loading, embedded structure, no detachment	High‐temperature structural damage, complex spinning, low yield, high cost	Porous electrodes, EM‐absorbing fibers, smart fibers	[95, 97]

Based on the aforementioned comparison, the selection of a preparation technique for Ti_3_C_2_T_x_ MXene‐modified CFs should involve a rational decision‐making process guided by the performance priorities of the target application. For high‐end composite materials pursuing ultimate interfacial enhancement and long‐term reliability (e.g., in aerospace or deep‐sea fields), chemical grafting should be prioritized due to its robust covalent bonding interface. When high electrical conductivity, EMI shielding, and efficient production are the core objectives, EPD (superior uniformity) and spray coating (high cost‐effectiveness and scalability) are more suitable for advanced electronic devices and large‐scale building materials. In the development of flexible wearable devices and smart textiles, self‐assembly technology stands out owing to its uniform film formation and ease of constructing flexible multilayer architectures. Blending‐spinning and carbonization, which enables intrinsic functionalization, represents a key technical pathway for exploring revolutionary integrations such as “structural batteries” and “structural sensors.” Furthermore, dip‐coating and drop‐casting, valued for their simplicity and rapidity, remain effective methods for laboratory‐scale material screening and prototype validation. In summary, there is no universally applicable “all‐in‐one” technique; precise process selection and potential hybrid approaches are crucial for optimizing material design. Future advancements will rely on the accurate selection and potential integration of techniques based on the target product's performance matrix (mechanical, electrical, durability, cost), thereby achieving optimal design of Ti_3_C_2_T_x_ MXene‐modified CFs.

## 3 Multifunctional Applications

### 3.1 Interfacial Enhancement

Owing to their outstanding comprehensive properties, CFRPs have achieved widespread application across numerous industrial sectors, establishing themselves as one of the most important materials of the 21st century. However, the inherent inertness of CF significantly limits interfacial bonding with polymer matrices, which remains a key bottleneck restricting both performance and application range. The use of Ti_3_C_2_T_x_ MXene nanoparticles offers a highly effective solution to interfacial challenges in CFRPs. The 2D lamellar structure of Ti_3_C_2_T_x_ MXene substantially increases the surface roughness of CFs, promoting mechanical interlocking with the matrix; meanwhile, its abundance of active surface functional groups improves wettability and facilitates the formation of hydrogen bonds and van der Waals interactions at the fiber‐matrix interface [99, 100]. As such, Ti_3_C_2_T_x_ MXene modification of CFs has been demonstrated as a powerful strategy to significantly enhance CFRP interfacial properties, making it a research hotspot in interfacial enhancement of CFRPs.

**FIGURE 7:**
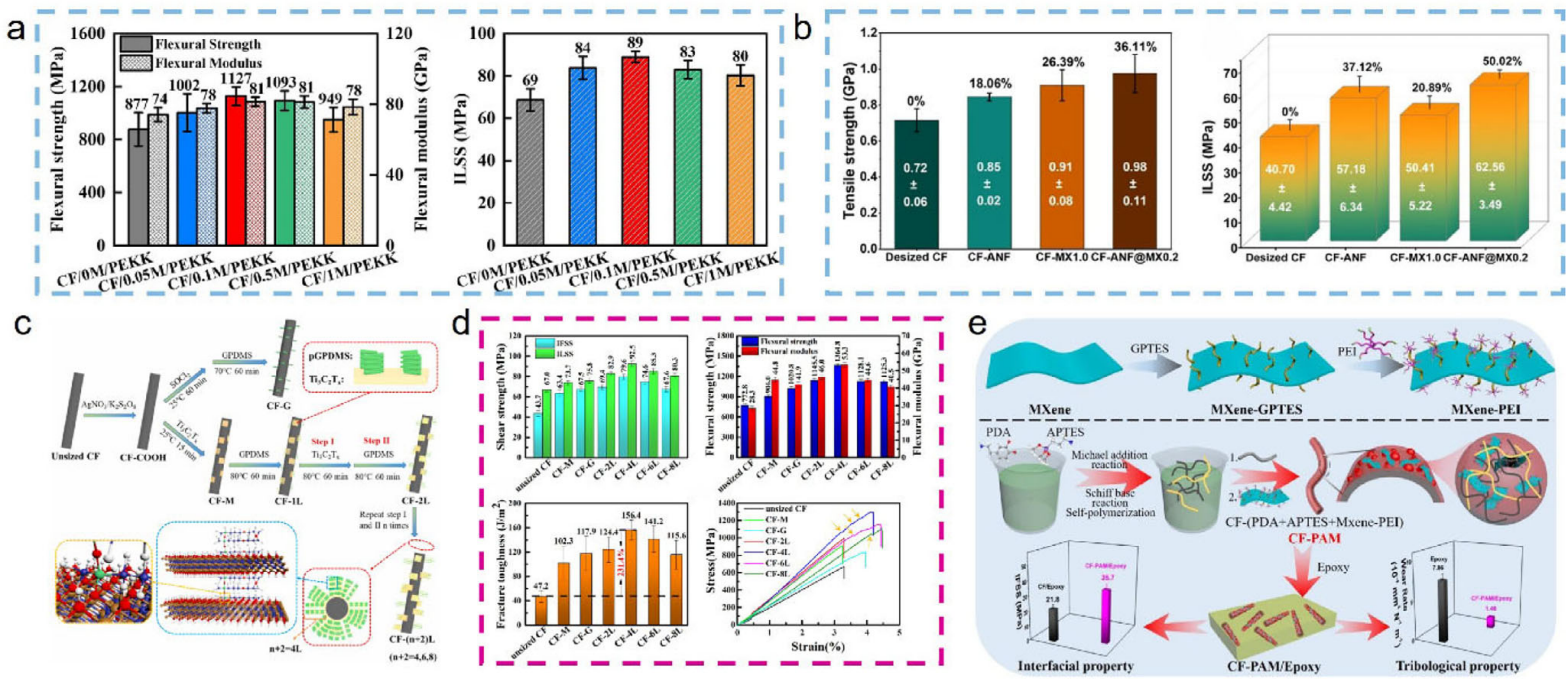
(a) Flexural strength, flexural modulus, and ILSS of CF/PEKK composites; Reproduced with permission [101]. Copyright 2023, Elsevier. (b) Tensile strength and ILSS of CF/PA composites; Reproduced with permission [35]. Copyright 2024, American Chemical Society. (c) Schematic illustration of Ti_3_C_2_T_x_ MXene‑modified CF prepared through LbL self‑assembly. (d) IFSS, ILSS, flexural strength, flexural modulus, fracture toughness, and stress‐strain curve of CF/EP composites. (c, d) Reproduced with permission [102]. Copyright 2023, Elsevier. (e) Fabrication of CF/EP composites with a “soft‐rigid” hybrid interphase and evaluation of their interfacial and tribological properties; Reproduced with permission [104]. Copyright 2025, Elsevier.

For example, Yang et al. [101]. prepared Ti_3_C_2_T_x_ MXene‐modified CFs via a dipping method, which significantly improved the flexural strength (1127 MPa), flexural modulus (81 GPa), and ILSS (89 MPa) of CF/polyetherketoneketone (PEKK) composites (Figure 7a). These values represent increases of 28.5%, 9.5%, and 29.7%, respectively, compared to unmodified CF/PEKK composites. The enhanced mechanical properties are mainly attributed to the introduction of Ti_3_C_2_T_x_ MXene, which induces effective mechanical interlocking between CF and PEKK, as well as the formation of hydrogen bonds and van der Waals forces, thereby significantly improving the interfacial adhesion and overall mechanical performance of the composites. Similarly, Zhang et al. [35]. used Ti_3_C_2_T_x_ MXene‐modified CFs in CF/polyamide (PA) composites, where the rigidity of Ti_3_C_2_T_x_ MXene sheets enhanced mechanical adhesion through physical anchoring, while Ti_3_C_2_T_x_ MXene's active functional groups promoted hydrogen bonding with oxygen‐containing groups in both the PA matrix and CFs. As a result, the ILSS and tensile strength of the composite reached 62.56 MPa and 0.98 GPa (Figure 7b), which are 50.02% and 36.11% higher than those of the unmodified CF/PA composites, respectively.

Moreover, the wealth of active sites on Ti_3_C_2_T_x_ MXene enables synergistic interactions with various modifiers, facilitating the introduction of stronger interfacial interactions, such as covalent and ionic bonds, and providing a versatile platform for designing hierarchical, multidimensional CFRP interface architectures [81]. For instance, Hu et al. [102]. employed a Layer‐by‐Layer (LbL) assembly of Ti_3_C_2_T_x_ MXene and GPDMS onto CFs, forming a multi‐layered hybrid interface between the CF and the EP matrix characterized by mechanical interlocking and covalent bonding (Figure 7c). Compared to the unmodified CF/EP composite, IFSS, flexural strength, flexural modulus, and fracture toughness were improved by 82.8%, 76.6%, 88.3%, and 231.4%, respectively (Figure 7d). Yuan et al. [103]. dip‐coated CFs with polyamide‐imide (PAI) and Ti_3_C_2_T_x_ MXene to generate a hierarchical transition layer at the CF/PEEK interface, featuring mechanical interlocking, hydrogen bonding, ionic bonds, and π‐π interactions. Consequently, compared to the unmodified CF/PEEK composite, flexural strength, flexural modulus, and ILSS increased by 52.49%, 112.51%, and 74.47%, respectively. Wang et al. [104]. created a “soft‐rigid” hybrid layer on the CF surface by grafting Ti_3_C_2_T_x_ MXene with PDA, APTES, and PEI (Figure 7e), which not only strengthened interactions with the epoxy matrix but also established a uniform and stable load transfer pathway within the interface layer, raising IFSS by 63.8% compared to the unmodified composite. The integration of hierarchical hybrid structures via Ti_3_C_2_T_x_ MXene modification thus opens unlimited possibilities for interfacial enhancement in CFRPs.

In summary, Ti_3_C_2_T_x_ MXene‐modified CFs deliver innovative solutions to overcome weak interfacial bonding in CFRPs. Future research should focus on investigating the long‐term interfacial stability under complex service conditions, developing scalable and environmentally‐friendly modification processes, and elucidating the reinforcement mechanisms of hierarchical interface architectures, thereby driving practical adoption of high‐performance CFRPs in aerospace, new energy equipment, and other advanced industries.

### 3.2 EMI Shielding

The highly ordered graphitic microcrystalline structure of CFs imparts them with excellent electrical properties, attracting widespread attention in the field of EMI shielding [105]. Meanwhile, the lightweight and high‐strength characteristics of CF grant it unique appeal in the current trend toward lightweight, flexible, and deformable EMI shielding materials [16]. As a result, CFRPs have become ideal candidates to replace metals in EMI shielding applications. However, the insulating polymer matrix between CF layers impedes vertical conduction pathways and significantly reduces electrical conductivity, which severely limits the EMI shielding performance of CFRPs. The application of Ti_3_C_2_T_x_ MXene‐modified CFs provides an effective solution to this problem.

**FIGURE 8:**
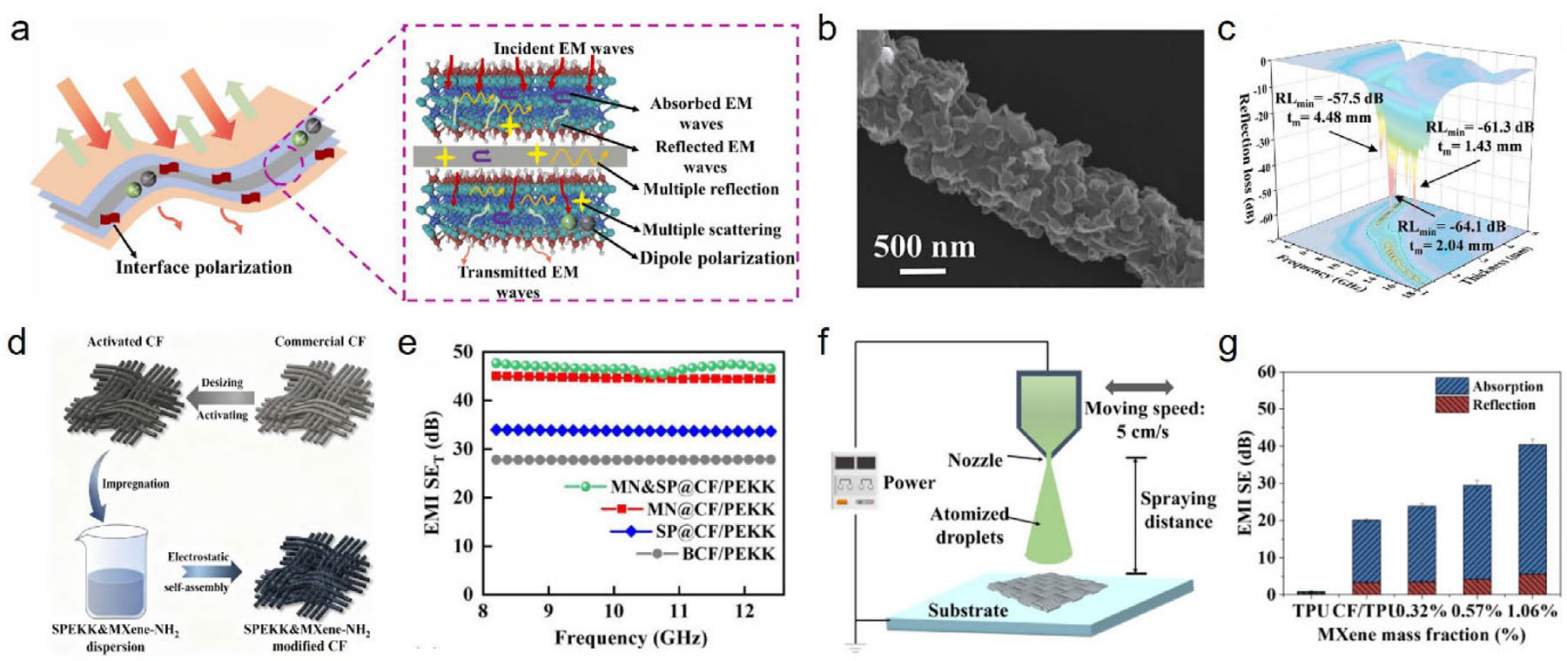
(a) EMI shielding mechanism of Ti_3_C_2_T_x_ MXene‐modified CF composites; Reproduced with permission [107]. Copyright 2023, Elsevier. (b) SEM image and (c) the EMW absorption performances of Ti_3_C_2_T_x_ MXene‐modified CF; Reproduced with permission [90]. Copyright 2025, Springer Nature. (d) Schematic diagram of the preparation process of Ti_3_C_2_T_x_ MXene‐modified CF through the self‑assembly process. (e) Total EMI SE of CF/PEKK composites. (d, e) Reproduced with permission [36]. Copyright 2025, Elsevier. (f) Schematic diagram of electrohydrodynamic atomization spraying of Ti_3_C_2_T_x_ MXene‐modified CF. (g) SE at various EMI shielding mechanisms (% absorption and % reflection) for different Ti_3_C_2_T_x_ MXene loading. (f, g) Reproduced with permission [113]. Copyright 2022, Elsevier.

Ti_3_C_2_T_x_ MXene‐modified CF primarily blocks EMI through two mechanisms: reflection, enabled by its high electrical conductivity, and absorption, where electromagnetic waves (EMWs) are dissipated as heat within its layered structure [106]. Figure 8a depicts the main mechanism underlying the EMI shielding performance of Ti_3_C_2_T_x_ MXene‐modified CF. Initially, due to impedance mismatch between free space and CFRP, part of the incident EMW is directly reflected, while the remainder penetrates the composite interior [38, 107]. Within the composite, these EMWs encounter the conductive network formed by Ti_3_C_2_T_x_ MXene nanosheets and CFs. High charge density regions in Ti_3_C_2_T_x_ MXene and CF, as well as the multiple conductive pathways provided, enable significant conductive losses and thus the dissipation of EMW energy [108, 109]. Additionally, the local dipoles formed by surface Ti atoms and terminal groups on Ti_3_C_2_T_x_ MXene enhance polarization loss, further increasing EMW attenuation [48, 110]. Interface polarization from the rich Ti_3_C_2_T_x_ MXene/CF interfaces, combined with dipole polarization of Ti_3_C_2_T_x_ MXene, generates substantial polarization loss and further EMW dissipation [111, 112]. Importantly, the hierarchical structure of Ti_3_C_2_T_x_ MXene‐modified CFs allows repeated EMW scattering and dissipation processes within CFRP, ensuring that EMWs are ultimately either absorbed or reflected [45, 103]. This multi‐hierarchical dissipation mechanism makes Ti_3_C_2_T_x_ MXene‐modified CFs a popular and widely utilized subject in EMI shielding studies.

For example, Feng et al. [90]. prepared 3D network‐structured Ti_3_C_2_T_x_ MXene‐modified CFs via electrospinning and carbonization (Figure 8b), achieving an RL_min_ of −64.1 dB and an EAB of 4.28 GHz for composites less than 2.1 mm thick, demonstrating excellent EMW absorption (Figure 8c). Yang et al. [36]. employed an electrostatic self‐assembly technique to sequentially assemble aminated Ti_3_C_2_T_x_ MXene and sulfonated PEKK onto CFs (Figure 8d). The resulting CF/PEKK composites exhibited an EMI SE of 44.6 dB, which is 60.4% higher than that of the unmodified CF/PEKK composites (Figure 8e), demonstrating outstanding EMI shielding performance. Duan et al. [113]. employed spray‐coating to deposit Ti_3_C_2_T_x_ MXene onto CFs (Figure 8f), followed by hot‐press molding with thermoplastic polyurethane (TPU). As the Ti_3_C_2_T_x_ MXene content increased, the EMI SE of the composites increased correspondingly, at a mass fraction of 1.06% (Figure 8g). Composites achieved an EMI SE of 40.4 dB at just 0.5 mm thickness, demonstrating remarkable EMI shielding performance.

In summary, Ti_3_C_2_T_x_ MXene nanoparticle, with its high electrical conductivity, 2D layered structure, and abundant active functional groups, can co‐construct efficient conductive networks with CF in polymer matrices. Meanwhile, it introduces various polarization loss mechanisms, such as interfacial polarization and dipole relaxation, into CFRP, thereby greatly enhancing the EMI shielding performance and injecting new vitality into the design of advanced EMI shielding materials. Future research should focus on optimizing the uniformity and orientation of Ti_3_C_2_T_x_ MXene distribution on the CF, as well as systematically investigating the synergistic shielding mechanisms among multiple components, to promote the practical application of these high‐performance EMI shielding materials in cutting‐edge fields such as 5G communications and wearable electronics.

### 3.3 High‐Efficiency Energy Storage

CF electrodes play a pivotal role in energy storage devices owing to their high electrical conductivity, chemical stability, lightweight nature, and flexible design capabilities. However, due to the inherently low specific surface area and reactivity of CFs, CFs often serve primarily as “conductive frameworks” onto which various electroactive materials are integrated, thereby enabling the fabrication of structural energy storage devices such as supercapacitors and batteries [114, 115]. Ti_3_C_2_T_x_ MXene is an exceptional modifier for CF electrodes, attributable to its Ti‐C backbone, which affords high electrical conductivity, a 2D layered structure that provides large specific surface area, and abundant redox‐active sites [116, 117].

**FIGURE 9:**
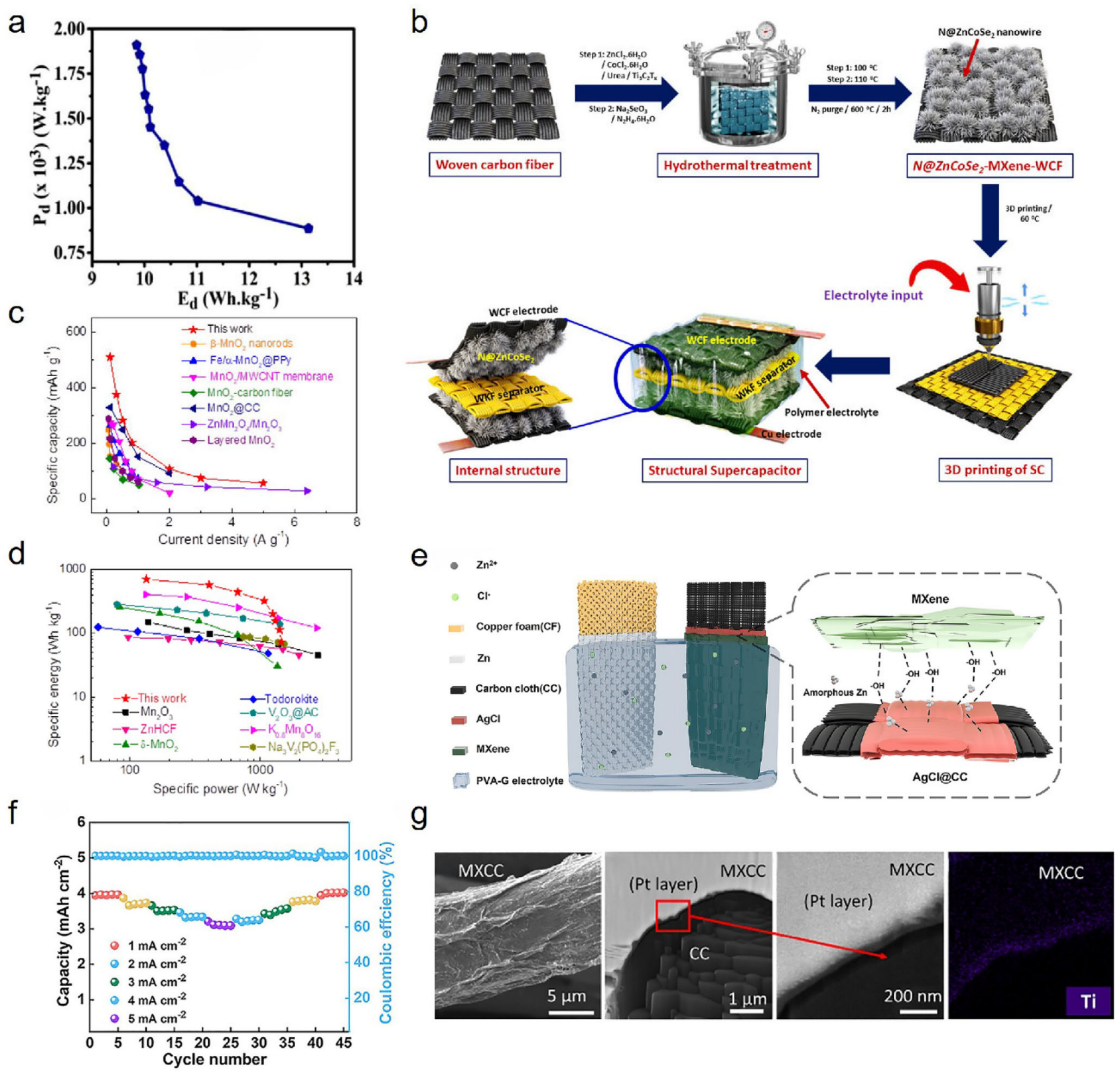
(a) Ragone plot of energy density vs. power density of Ti_3_C_2_T_x_ MXene‐modified CF flexible supercapacitors; Reproduced with permission [118]. Copyright 2023, Elsevier. (b) Fabrication schematic of the N‑doped zinc‐cobalt selenide nanowire/Ti_3_C_2_T_x_ MXene‑modified CF electrode and the corresponding supercapacitor device; Reproduced with permission [119]. Copyright 2023, American Chemical Society. (c) The rate performance and (d) Ragone plot of Ti_3_C_2_T_x_ MXene/MnO_2_‐modified CF; Reproduced with permission [120]. Copyright 2022, Elsevier. (e) Schematic illustration of Ti_3_C_2_T_x_ MXene‐modified AgCl/CF cathode. (f) Discharge characteristics and Coulombic efficiency of the battery with Ti_3_C_2_T_x_ MXene‐modified AgCl/CF cathode at various current densities. (e, f) Reproduced with permission [121]. Copyright 2023, Elsevier. (g) Cross‐sectional SEM image of the Ti_3_C_2_T_x_ MXene‐modified CF and the corresponding Ti elemental mapping obtained by EDS; Reproduced with permission [122]. Copyright 2024, American Chemical Society.

To date, Ti_3_C_2_T_x_ MXene‐modified CF electrodes have exhibited excellent electrochemical efficiency and capacitance, finding wide application as electrodes in supercapacitors and other energy storage devices. For example, Singh et al. [118]. fabricated Ti_3_C_2_T_x_ MXene‐modified CF electrodes via drop‐casting, flexible supercapacitors assembled from these electrodes displayed a maximum energy density of 13.3 Wh·kg^−1^ and a peak power density of 1.91 kW·kg^−1^ (Figure 9a). The electrochemical characteristics of Ti_3_C_2_T_x_ MXene‐modified CF electrodes make them ideal candidates for flexible energy storage devices. Deka et al. [119]. fabricated an N‐doped zinc‐cobalt selenide nanowire/Ti_3_C_2_T_x_ MXene‐modified CF electrode and employed it in a thermosetting polymer electrolyte‐based structural supercapacitor (Figure 9b). The device achieved a specific capacitance of 19.36 F g^−1^, at a current density of 1000 mA g^−1^, it delivered an energy density of 2.69 Wh kg^−1^ and a power density of 43.20 W kg^−1^. The incorporation of Ti_3_C_2_T_x_ MXene endowed the device with enhanced stability, rate capability, and redox potential. Qi et al. [120]. prepared flexible Ti_3_C_2_T_x_ MXene/MnO_2_‐modified CF electrodes by a dipping method, when used as the cathode material for zinc‐ion batteries, these electrodes demonstrated high charge storage performance (517.0 mAh g^−1^ at 0.1 A g^−1^) (Figure 9c), outstanding energy density (701.3 Wh kg^−1^ at 133.8 W kg^−1^) (Figure 9d), and excellent cycling stability (80.6 mAh g^−1^ after 800 cycles at 1 A g^−1^). The highly conductive Ti_3_C_2_T_x_ MXene functions not only as a stable conductive scaffold but also provides additional Zn^2+^ storage sites, thereby enhancing the overall charge storage capability.

In addition, Ti_3_C_2_T_x_ MXene‐modified CF electrodes demonstrate impressive stability and durability, as well as effective inhibition of corrosion and dendrite formation. Zhu et al. [121]. utilized drop‐casting to produce Ti_3_C_2_T_x_ MXene‐modified AgCl/CF cathode materials for flexible Ag‐Zn batteries (Figure 9e). Uniform Ti_3_C_2_T_x_ MXene coating maintained electrode integrity, suppressed Ag loss, and endowed the CF electrode with an ultra‐high surface area, resulting in a battery with a high areal capacity of 2.97 mAh·cm^−2^ and nearly 100% coulombic efficiency after 400 cycles at 4 mA·cm^−2^ (Figure 9f). De Alwis et al. [122]. prepared Ti_3_C_2_T_x_ MXene‐modified CF electrodes for zinc‐ion battery anodes via coating, where a 30 nm Ti_3_C_2_T_x_ MXene layer enhanced Zn^2+^ adhesion and diffusion kinetics (Figure 9g), suppressed dendrite growth and anode corrosion, and enabled the full cell to achieve a high capacity of 260 mAh·g^−1^ at 0.25 A·g^−1^, with cycle life extending to 1000 cycles.

In summary, Ti_3_C_2_T_x_ MXene‐modified CF electrodes, through the synergistic combination of the macroscopic conductive framework of CF and the nanoscale electrochemical activity of Ti_3_C_2_T_x_ MXene, provide innovative solutions for next‐generation high‐performance energy storage devices. Future research should address the structural stability of Ti_3_C_2_T_x_ MXene during long‐term cycling and explore its potential in diverse energy storage systems, thus advancing the industrial application of flexible energy storage devices.

### 3.4 Emerging Application Scenarios

With rapid technological development and industrial upgrading in the last decade, the immense potential of Ti_3_C_2_T_x_ MXene‐modified CFs is no longer confined to traditional fields such as interfacial reinforcement, EMI shielding, and energy storage. Through sophisticated surface engineering and structural design, Ti_3_C_2_T_x_ MXene‐modified CFs not only significantly enhance the intrinsic properties of CFs but also impart novel features such as high electrical conductivity, excellent electrochemical activity, and outstanding photothermal conversion. This offers innovative solutions for frontier applications in sensing, thermal management, catalysis, and more.

#### 3.4.1 Intelligent Sensing

Intelligent sensing constitutes the core foundation for emerging fields such as health monitoring, the Internet of Things, and human‐machine interaction, and is rapidly evolving toward higher sensitivity, multifunctional integration, flexibility, and self‐powered capabilities. The development of high‐performance sensing materials is essential for driving advancements in this domain [123]. Owing to their unique composition and structural characteristics, Ti_3_C_2_T_x_ MXene‐modified CFs present highly promising prospects for the research and enhancement of next‐generation intelligent sensors.

**FIGURE 10:**
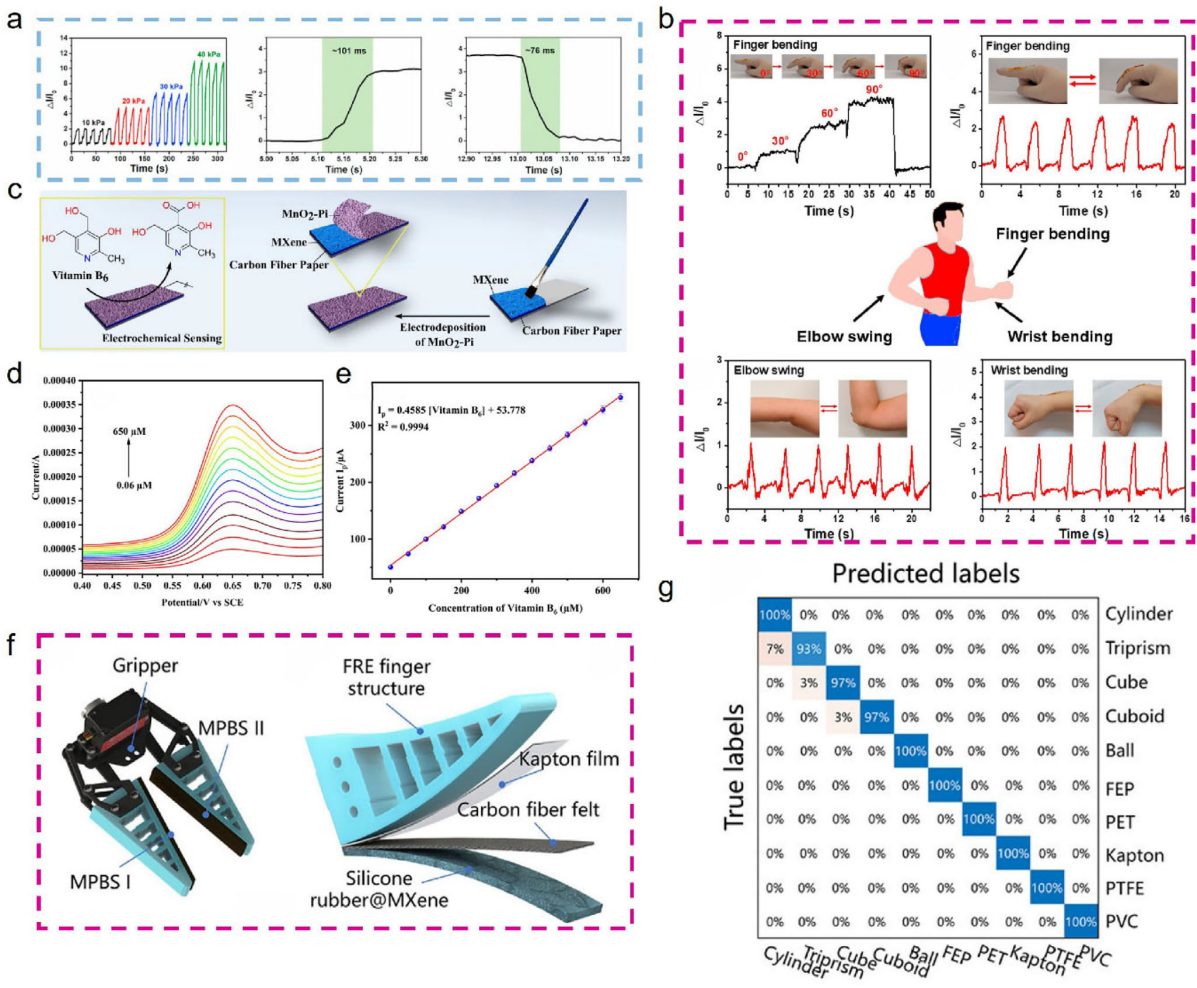
(a) Dynamic response situation of the sensor during the cyclic loading‐unloading process under different pressures. (b) Applications of the sensor for real‐time monitoring of human motions, the relative current changes come from finger bending, elbow swing, and wrist bending. (a, b) Reproduced with permission [125]. Copyright 2022, Elsevier. (c) Schematic illustration of the fabrication of Ti_3_C_2_T_x_ MXene‑modified CF electrodes for electrochemical detection of Vitamin B_6_. (d) Linear detection range of Vitamin B_6_ and (e) calibration curve used for limit‑of‑detection calculation of Ti_3_C_2_T_x_ MXene‑modified CF electrode. (c–e) Reproduced with permission [127]. Copyright 2023, Elsevier. (f) Bimodal triboelectric sensor based on Ti_3_C_2_T_x_ MXene‑modified CFs, integrated into a soft robotic gripper for object recognition. (g) Confusion map of the system recognizing mixed information about objects, total accuracy: 98.7%. (f, g) Reproduced with permission [41]. Copyright 2025, Wiley.

The multilevel layered structure of Ti_3_C_2_T_x_ MXene enables pronounced elastic deformation, under varying pressures, the interlayer spacing changes, resulting in modulated resistance values that are transmitted as electrical signals along the conductive pathways of MXene‐modified CFs, enabling high‐sensitivity pressure sensing [31, 124]. For example, Wang et al. [125]. fabricated Ti_3_C_2_T_x_ MXene‐modified CF/PDMS composites via a dipping method for pressure sensing applications. The sensor exhibited fast and stable responses during cyclic loading and unloading under different pressures, with response and recovery times of approximately 101 and 76 ms, respectively (Figure 10a). In their study, the device accurately tracked various movements of different limbs (Figure 10b), demonstrating significant potential for real‐time motion and health monitoring applications.

Moreover, Ti_3_C_2_T_x_ MXene‐modified CFs are ideal for electrochemical sensor fabrication. The high surface area and abundance of active sites in Ti_3_C_2_T_x_ MXene provide an excellent platform for molecular recognition and detection, while the flexibility and conductivity of CFs make them attractive candidates for sensor electrodes [126]. For instance, Rajeev et al. [127]. coated CF electrodes with Ti_3_C_2_T_x_ MXene for the quantitative detection of Vitamin B_6_ (Figure 10c), producing an electrochemical sensor with a wide linear detection range (0.06–650 µM) and an ultra‐low detection limit of 0.021 µM (Figure 10d,e), indicating significant value for health monitoring and clinical diagnostics. Recent studies have further demonstrated the high sensitivity and low detection limits of Ti_3_C_2_T_x_ MXene‐modified CF in electrochemical sensing of molecules such as KA [65], miRNA [128], glucose [129], uric acid [130], and more, suggesting its great potential as a universal platform for electrochemical sensing applications.

With ongoing research, the pursuit of highly integrated and self‐powered intelligent sensing devices has become a major trend, and Ti_3_C_2_T_x_ MXene‐modified CFs also show substantial potential in this arena. The high electrical conductivity and negatively charged surface of Ti_3_C_2_T_x_ MXene‐modified CF endow it with remarkable electrical output and charge‐capturing capabilities, which enable it to serve not only as a flexible electrode but also as a triboelectric layer, powering sensors themselves or other microelectronic devices, while realizing triboelectric sensing through contact electrification and electrostatic induction with external objects [131, 132]. For example, Dong et al. [41]. fabricated a bimodal triboelectric sensor using Ti_3_C_2_T_x_ MXene‐modified CFs (Figure 10f), which not only provided self‐powering for robotic arms but also dramatically improved the perception and actuation performance of soft robots, enabling accurate discrimination, stable grasping, and precise characterization of objects with an accuracy rate as high as 98.7% (Figure 10g).

In summary, by harnessing the complementary microstructural and functional advantages, Ti_3_C_2_T_x_ MXene‐modified CF has enabled the construction of a new generation of high‐performance intelligent sensing platforms. Its remarkable potential in flexible physical sensing, chemical/biological detection, and self‐powered system integration suggests a transformative impact on healthcare, biochemical monitoring, intelligent robotics, and more. Future research should focus on enhancing long‐term stability in complex environments, optimizing signal consistency and reproducibility, exploring integration with flexible electronic systems, and strengthening applied validation in relevant fields to promote the advancement of intelligent sensing technologies toward greater efficiency and intelligence.

#### 3.4.2 Thermal Management

Thermal management has become a critical challenge in modern industries, particularly in fields such as electronic packaging, aerospace, advanced energy storage systems, and wearable devices, where efficient collection, conduction, and insulation of heat are essential [133]. During the past few years, CFs have emerged as ideal candidates for thermal management composites due to their excellent mechanical properties and inherent thermal conductivity [134]. Meanwhile, Ti_3_C_2_T_x_ MXene, thanks to its outstanding metallic conductivity, intrinsically high thermal conductivity, and exceptional electrothermal and photothermal conversion efficiencies, offers new design strategies for next‐generation high‐performance thermal management composites [135].

**FIGURE 11:**
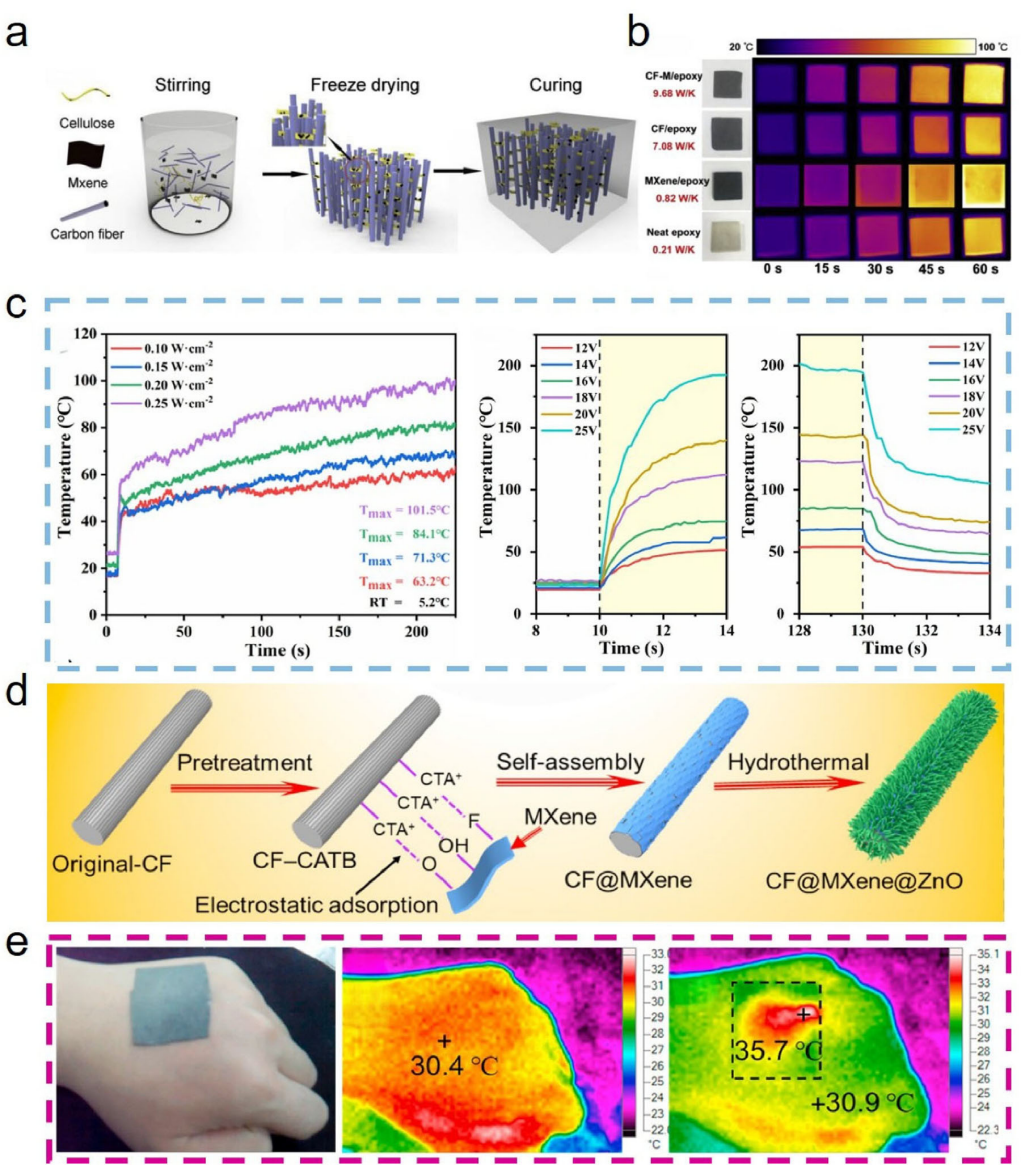
(a) The preparation process of directionally customized Ti_3_C_2_T_x_ MXene‐modified CF/EP composites. (b) Infrared images of CF/EP composites during heating. (a, b) Reproduced with permission [136]. Copyright 2020, Elsevier. (c) Temperature‐time curves of the Ti_3_C_2_T_x_ MXene‐modified CF paper under different light irradiation densities; Reproduced with permission [32]. Copyright 2022, Elsevier. (d) Schematic diagram of the preparation process of Ti_3_C_2_T_x_ MXene/ZnO‐modified CFs through the self‑assembly process. (e) IR thermal images of a hand with the composite before and after light irradiation for 2 min. (d, e) Reproduced with permission [137]. Copyright 2021, Elsevier.

By synergistically integrating the advantages of both materials, Ti_3_C_2_T_x_ MXene‐modified CFs have enabled significant improvements in thermal collection, transfer, and protection properties of composites. For example, Guo et al. [136]. developed directionally customized Ti_3_C_2_T_x_ MXene‐modified CF/EP composites through a freeze‐drying process (Figure 11a), the resulting material exhibited an ultrahigh thermal conductivity of 9.68 W/mK, which represents a 36.7% improvement over conventional CF/EP composites (Figure 11b). In addition, this composite demonstrated an extremely low coefficient of thermal expansion, making it an ideal thermal management material for electronic packaging applications. Wang et al. [32]. prepared Ti_3_C_2_T_x_ MXene‐modified CF/PDMS composites with remarkable electrothermal conversion capability. Under a 25 V applied voltage, the composite's temperature could exceed 200°C (Figure 11c), meeting requirements for wearable thermal management and even large‐scale equipment de‐icing. Additionally, Han et al. [137]. synthesized core‐sheath structured Ti_3_C_2_T_x_ MXene/ZnO‐modified CFs via electrostatic self‐assembly and incorporated them into CF/polyurethane (PU) flexible composites (Figure 11d). These materials exhibited outstanding photothermal conversion; after 2 min of illumination, the temperature of the composite material increased by 5.3°C, while the unexposed part only rose by 0.5°C (Figure 11e), demonstrating excellent photothermal conversion capability and suggesting direct application potential as thermal insulation material for wearable devices. Of note, Ti_3_C_2_T_x_ MXene‐modified CFs exhibit rapid thermal response, high cycling stability, a wide range of tunable operating temperatures, and excellent durability, thereby laying a solid foundation for their application in next‐generation thermal management materials [32, 108].

In summary, Ti_3_C_2_T_x_ MXene‐modified CFs demonstrate exceptional integrated performance and broad application potential in thermal management. The effective combination of the unique properties of Ti_3_C_2_T_x_ MXene and CF not only meets the stringent requirements for high thermal conductivity and low thermal expansion in electronic packaging but also provides innovative solutions for thermal comfort regulation in wearable devices and active thermal protection in large‐scale equipment. Future research should focus on optimizing interfacial thermal resistance through strategies like interface modifier design and surface terminal group regulation to reduce phonon scattering, enhancing long‐term thermal stability via approaches such as protective encapsulation and compounding with oxidation‐resistant materials, and further refining structural design and fabrication processes, particularly the development of continuous processes for constructing controlled 3D thermal networks. Additionally, it is essential to explore their practical potential in demanding scenarios such as aerospace thermal protection and power battery thermal management, thus providing a new materials basis for advances in high‐efficiency thermal management technologies.

#### 3.4.3 Catalysis

With the growing focus on clean energy and green synthesis, high‐efficiency and stable catalysts are increasingly in demand, bringing the catalytic applications of Ti_3_C_2_T_x_ MXene‐modified CFs into sharp research attention. The unique 2D structure, abundant active sites, and superior electron transport capacity of Ti_3_C_2_T_x_ MXene effectively overcome the inherent inertness and limited active sites of CF surfaces; at the same time, they harness CF's structural robustness and electrical conductivity, providing high‐performance platforms for various catalytic reactions [129, 138].

**FIGURE 12:**
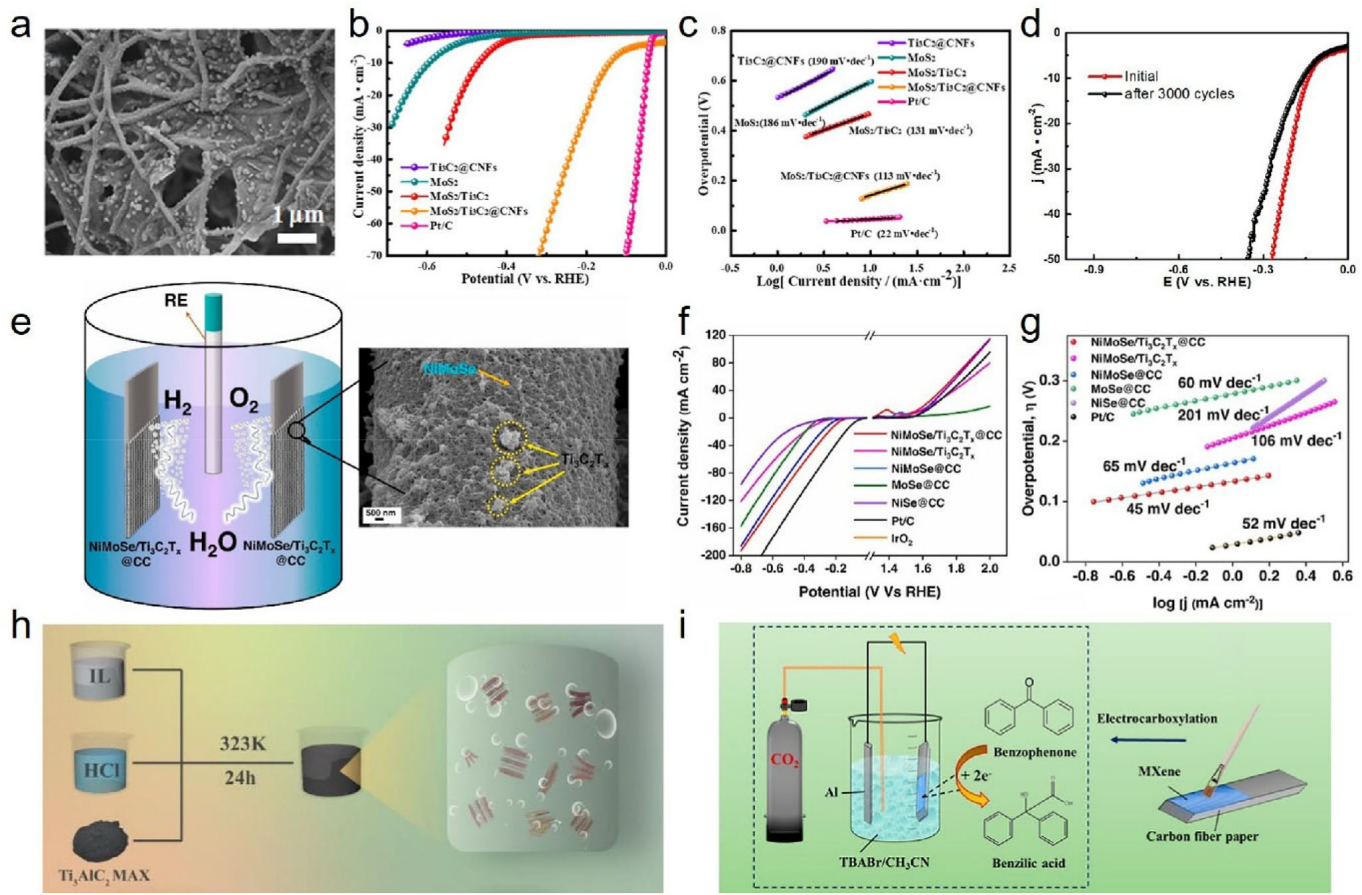
(a) SEM image of MoS_2_/Ti_3_C_2_T_x_ MXene‐modified CFs. (b) Polarization curves and (c) Tafel slopes of CFs. (d) Long‐term durability test of MoS_2_/Ti_3_C_2_T_x_ MXene‐modified CFs. (a–d) Reproduced with permission [39]. Copyright 2022, Elsevier. (e) Schematic diagram of NiMoSe/Ti_3_C_2_T_x_ MXene‑modified CFs for catalyzing oxygen and hydrogen evolution reaction. (f) Polarization curves and (g) Tafel slopes of CFs. (e–g) Reproduced with permission [139]. Copyright 2024, Elsevier. (h) Schematic illustration of the ionic‑liquid etching process of Ti_3_C_2_T_x_ MXene; Reproduced with permission [140]. Copyright 2022, Elsevier. (i) Schematic illustration of Ti_3_C_2_T_x_ MXene‑modified CF electrode for catalyzing electrocarboxylation reaction; Reproduced with permission [142]. Copyright 2024, American Chemical Society.

Ti_3_C_2_T_x_ MXene‐modified CFs have shown promise as electrocatalysts for hydrogen/oxygen evolution reactions in water splitting. For example, Ma et al. [39]. fabricated MoS_2_/Ti_3_C_2_T_x_ MXene‐modified CFs for the electrocatalytic hydrogen evolution reaction. The modified CF exhibited a high nanoparticle loading capacity and significantly promoted electron transport (Figure 12a). At a current density of 10 mA cm^−2^, it delivered a low overpotential of 142 mV and a Tafel slope of only 113 mV·dec^−1^ (Figure 12b,c). Furthermore, the CF maintained high catalytic activity even after 3000 cycles (Figure 12d), indicating that Ti_3_C_2_T_x_ MXene‐modified CF possesses excellent catalytic activity and chemical stability during the hydrogen evolution reaction. Saquib et al. [139]. developed a NiMoSe/Ti_3_C_2_T_x_ MXene‐modified CF with a highly dense nanoporous structure for electrocatalysis (Figure 12e). The modified CF exhibited high electrocatalytic activity for both hydrogen and oxygen evolution reactions, with a dual‐function catalyst showing an overpotential of 203 mV and a Tafel slope of 45 mV·dec^−1^ (Figure 12f,g). Beyond water splitting, Ti_3_C_2_T_x_ MXene‐modified CFs also show strong potential for catalyzing redox reactions of electrolyte species. Jing et al. [140]. modified CFs with Ti_3_C_2_T_x_ MXene etched using ionic liquids and HCl (Figure 12h), resulting in electrodes that dramatically enhanced V^3+^/V^2+^ redox catalysis, with the diffusion coefficient and electrochemical rate constant for the anodic oxidation reaction two orders of magnitude higher than those of unmodified CF, indicating exceptional electrochemical and catalytic activities.

Moreover, Ti_3_C_2_T_x_ MXene‐modified CFs are opening new possibilities in catalytic conversion and fixation of greenhouse gases and in organic synthesis [141]. For instance, Sariga et al. [142]. utilized Ti_3_C_2_T_x_ MXene‐modified CFs to catalyze the electrocarboxylation of benzophenone to diphenylacetic acid in a CO_2_ atmosphere (Figure 12i), achieving a 72% yield. The high electrochemical activity and excellent electrocatalytic ability of the Ti_3_C_2_T_x_ MXene‐modified CFs promoted mass transport and reaction kinetics during benzophenone electrocarboxylation, providing a highly efficient catalytic system for greenhouse gas utilization and green synthesis.

In summary, Ti_3_C_2_T_x_ MXene‐modified CFs, benefiting from their unique structure and performance attributes, exhibit broad prospects in the field of catalysis. However, research remains at an early stage. Future developments should address long‐term catalytic activity by fundamentally understanding deactivation mechanisms and designing stabilized structures (e.g., via protective coatings or doping), scalable fabrication technologies centered on continuous processes like electrochemical or spray deposition, and recyclability strategies focused on enabling facile catalyst separation and regeneration. Concurrently, expanding the application scope to areas such as gas‐phase heterogeneous catalysis (e.g., for pollutant degradation) and solar‐driven photoelectrocatalysis will be essential to fully exploit the multifunctional platform. It is anticipated that Ti_3_C_2_T_x_ MXene‐modified CFs will provide next‐generation high‐efficiency platforms for clean energy conversion and sustainable chemical synthesis, thereby advancing the progress toward green and sustainable chemistry.

#### 3.4.4 Safety Protection

With the growing application of CFs and their composites across diverse fields, their performance under extreme conditions remains a critical concern [143, 144]. In practice, CFRPs often exhibit unsatisfactory behavior when confronted with hazards such as fire and lightning strikes. The recent advent of Ti_3_C_2_T_x_ MXene‐modified CFs offers a promising strategy to enhance the safety and protection properties of CFRPs.

**FIGURE 13:**
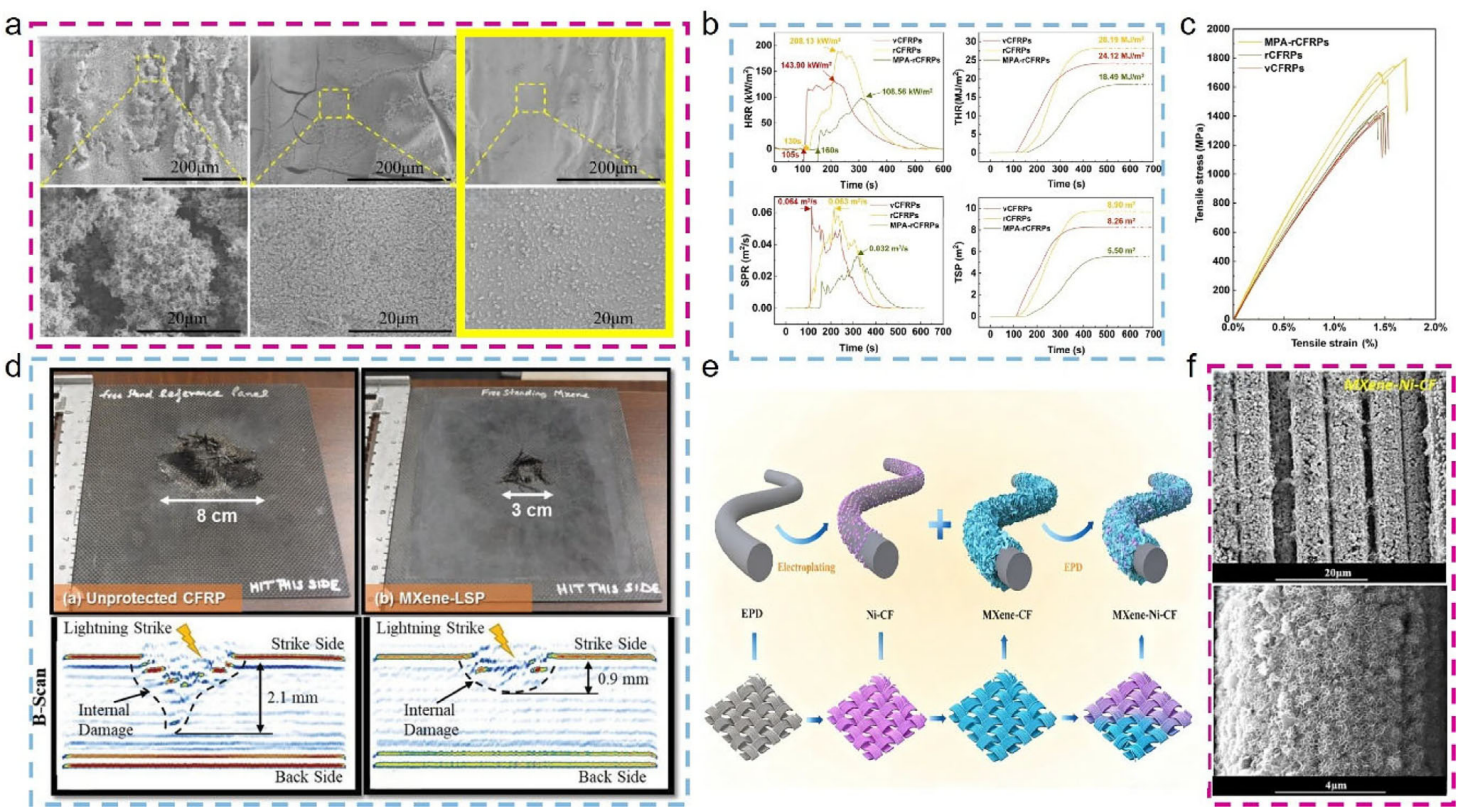
(a) SEM images of the external char residues of CF/PVA aerogel composites, where the Ti_3_C_2_T_x_ MXene‑modified CF/PVA aerogel composite (yellow marker) exhibits a dense structure conducive to flame suppression; Reproduced with permission [146]. Copyright 2022, Elsevier. (b) Results of cone calorimetric test and (c) tensile strain and stress curves of CFRPs; Reproduced with permission [48]. Copyright 2025, Elsevier. (d) Damage response images of CFRP panels after lightning strike experiments; Reproduced with permission [147]. Copyright 2021, Wiley. (e) Schematic image of the preparation processes of Ti_3_C_2_T_x_ MXene/Ni‐modified CFs via the electroplating and EPD technique. (f) SEM images of Ti_3_C_2_T_x_ MXene/Ni‐ modified CFs. (e, f) Reproduced with permission [40]. Copyright 2023, Elsevier.

Recent studies have shown that Ti_3_C_2_T_x_ MXene modification significantly improves the flame retardancy of CFRPs. Under high temperatures, Ti_3_C_2_T_x_ MXene is oxidized to form substantial carbon layers and TiO_2_ particles, which together construct a stable, dense physical barrier that effectively suppresses combustion [145]. For instance, Zhou et al. [146]. fabricated Ti_3_C_2_T_x_ MXene‐modified CF/PVA aerogel composites; cone calorimetry tests and post‐combustion char morphology analyses confirmed markedly improved flame retardancy, smoke suppression, and toxic gas inhibition (Figure 13a). Similarly, Hu et al. [48]. demonstrated that their Ti_3_C_2_T_x_ MXene‐modified CF composites exhibit superior thermal stability, with calorimetry indicating that the carbon and rigid TiO_2_ layers formed upon oxidation act as effective barriers to thermal transfer and smoke release (Figure 13b). Notably, unlike traditional flame‐retardant fillers introduced into the CFRP matrix, Ti_3_C_2_T_x_ MXene‐modified CFs ensure more uniform stress distribution within the composite, thereby enhancing flame retardancy without compromising the mechanical integrity of CFRPs (Figure 13c).

In addition, the highly conductive network formed between Ti_3_C_2_T_x_ MXene and CF allows for rapid dissipation of incident currents, enabling application in lightning strike protection. For example, Kumar et al. [147]. coated the surface of CFRP with Ti_3_C_2_T_x_ MXene to monitor its lightning strike protection performance. Results from 100 kA lightning strike tests showed that Ti_3_C_2_T_x_ MXene‐modified CFRP has a significantly smaller damage area and penetration depth than unmodified CFRP (Figure 13d), confirming that Ti_3_C_2_T_x_ MXene can significantly enhance the lightning protection capability of CFRP. Hu et al. [40]. utilized cathodic EPD and electroplating to uniformly distribute Ti_3_C_2_T_x_ MXene and Ni on CFs (Figure 13e), achieving in‐plane and through‐thickness conductivities of 195,350 S/m and 4,570 S/m, corresponding to 14‐fold and 116‐fold increases, respectively. Such uniform Ti_3_C_2_T_x_ MXene/Ni distribution is critical for efficient electron transfer and, by extension, significantly enhances CFRP lightning strike resistance (Figure 13f).

In summary, Ti_3_C_2_T_x_ MXene‐modified CFs provide an important pathway to enhance the performance of CFRPs in extreme environments. To translate this potential into practical engineering solutions, future research must strategically address several multi‐scale challenges. A primary focus should be on advanced techniques that guarantee the uniform and conformal coating of Ti_3_C_2_T_x_ MXene on CFs and complex preform surfaces, as local defects or inhomogeneities can become failure origins. Concurrently, a systematic investigation into the long‐term stability and degradation mechanisms under coupled extreme conditions is crucial for predictive lifetime modeling. Furthermore, the processing and preparation of large‐scale CF protective materials require further optimization to enhance production efficiency and performance assurance, thereby bridging the gap between actual production and laboratory preparation. Success in these areas will be foundational to delivering a new generation of safer, more durable, and multifunctional high‐performance composites for mission‐critical applications in next‐generation aerospace, new energy vehicles, and advanced marine engineering.

#### 3.4.5 Optoelectronic Devices

With the increasing importance of micro‐ and nano‐electronic devices and the photonics industry, Ti_3_C_2_T_x_ MXene‐modified CFs display tremendous application potential for optoelectronic devices. The large specific surface area, high photoresponsivity, and excellent conductivity of Ti_3_C_2_T_x_ MXene confer Ti_3_C_2_T_x_ MXene‐modified CFs with high carrier transport efficiency, which in turn can significantly enhance the sensitivity and photo‐detection response speed of optoelectronic devices. Additionally, Ti_3_C_2_T_x_ MXene exhibits ultra‐wideband light absorption spanning a broad wavelength range [148, 149]. By integrating the respective advantages of Ti_3_C_2_T_x_ MXene and CF, these composites are particularly well suited for applications in flexible electronics and optoelectronic sensing.

**FIGURE 14:**
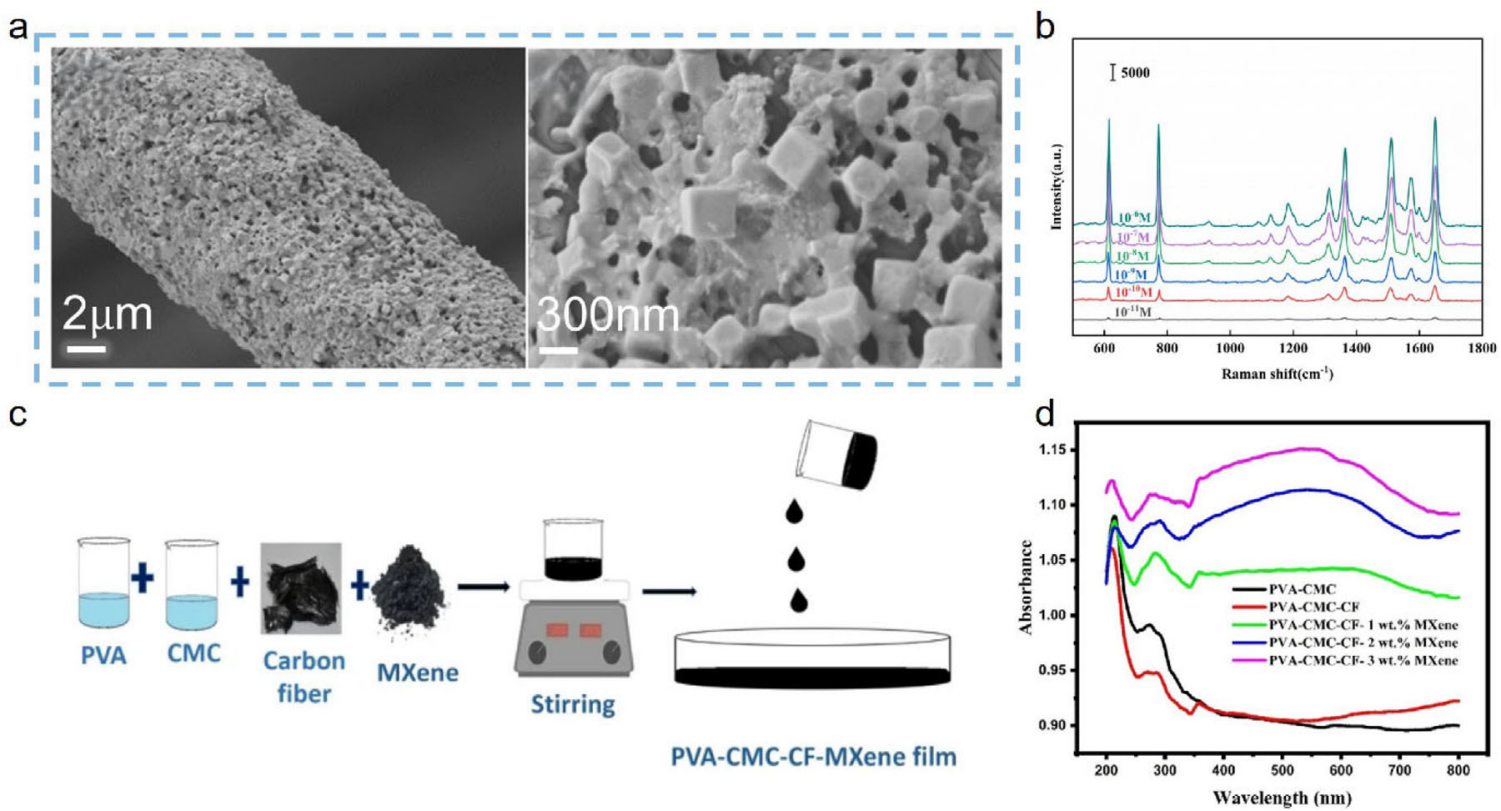
(a) SEM images of ZIF‐67/Ti_3_C_2_T_x_ MXene‐modified CF. (b) Raman signals of rhodamine 6G at different concentration gradients. (a, b) Reproduced with permission [150]. Copyright 2025, Royal Society of Chemistry. (c) Schematic diagram of preparation of Ti_3_C_2_T_x_ MXene‐modified CF/PVA/CMC composites. (d) UV absorption spectra of different Ti_3_C_2_T_x_ MXene‐modified CF/PVA/CMC composites. (c), d Reproduced with permission [151]. Copyright 2025, Springer Nature.

Although the use of Ti_3_C_2_T_x_ MXene‐modified CFs in optoelectronic devices is still in an early research stage, their unique properties remain highly attractive. For example, Shao et al. [150]. fabricated ZIF‐67/Ti_3_C_2_T_x_ MXene‐modified CFs as surface‐enhanced Raman scattering (SERS) substrates. The SEM images of ZIF‐67/Ti_3_C_2_T_x_ MXene‐modified CF are shown in Figure 14a. The ZIF‐67/Ti_3_C_2_T_x_ MXene heterojunction ensured the Ti_3_C_2_T_x_ MXene's high stability and optoelectronic performance, while the flexibility of CF supported the detection of irregularly shaped samples. Remarkably, the device enabled in situ detection of pesticide residues on apple surfaces with an ultra‐low detection limit (10^−11^ M), exemplifying the unique advantages of Ti_3_C_2_T_x_ MXene‐modified CFs (Figure 14b). Atta et al. [151]. prepared and evaluated the optical properties of Ti_3_C_2_T_x_ MXene‐modified CF/PVA/CMC composites (Figure 14c). They found that increasing MXene content led to a surge in free electron concentration and a pronounced increase in UV‐visible absorbance (Figure 14d), making these materials promising candidates for high‐speed communication, UV shielding, and fast optical switching.

In summary, Ti_3_C_2_T_x_ MXene‐modified CFs demonstrate distinctive advantages in optoelectronic devices and are poised to serve as core materials for the next generation of photonic and electronic technologies, including flexible electronics, transparent devices, and wearable systems. Future research should focus on several critical challenges. Paramount among these is ensuring long‐term material reliability under operational stresses (e.g., bending, environmental exposure), and advancing interface band structure engineering (e.g., optimizing carrier transport and photoresponse properties through surface functional group modulation, heterostructure construction, or doping modification). Furthermore, exploring sophisticated heterojunction strategies with other low‐dimensional nanomaterials (e.g., perovskites, 2D transition metal dichalcogenides) will be key to enabling novel charge‐transfer pathways and achieving high‐performance, multifunctional integration in next‐generation flexible optoelectronic devices.

#### 3.4.6 Biomedical Applications

CFs are widely employed in biomedical applications such as bone repair scaffolds, neural electrodes, and ligament substitutes due to their high specific strength, excellent chemical stability, electrical conductivity, and modulus similar to biological tissue. However, their inherently bioinert surfaces limit cell adhesion, differentiation, and proliferation, ultimately restricting efficient integration with biological tissues [152, 153]. More recently, Ti_3_C_2_T_x_ MXene has garnered considerable attention as an advanced candidate for engineering traditional biomaterials, attributed to its outstanding electrical conductivity, rich surface functionalities, pronounced hydrophilicity, facile chemical tailoring capability, and well‑established biocompatibility [154]. Constructing Ti_3_C_2_T_x_ MXene‐modified CFs effectively capitalizes on the strengths of both materials, overcoming CF's bioinertness and significantly expanding its applicability in biomedicine.

**FIGURE 15:**
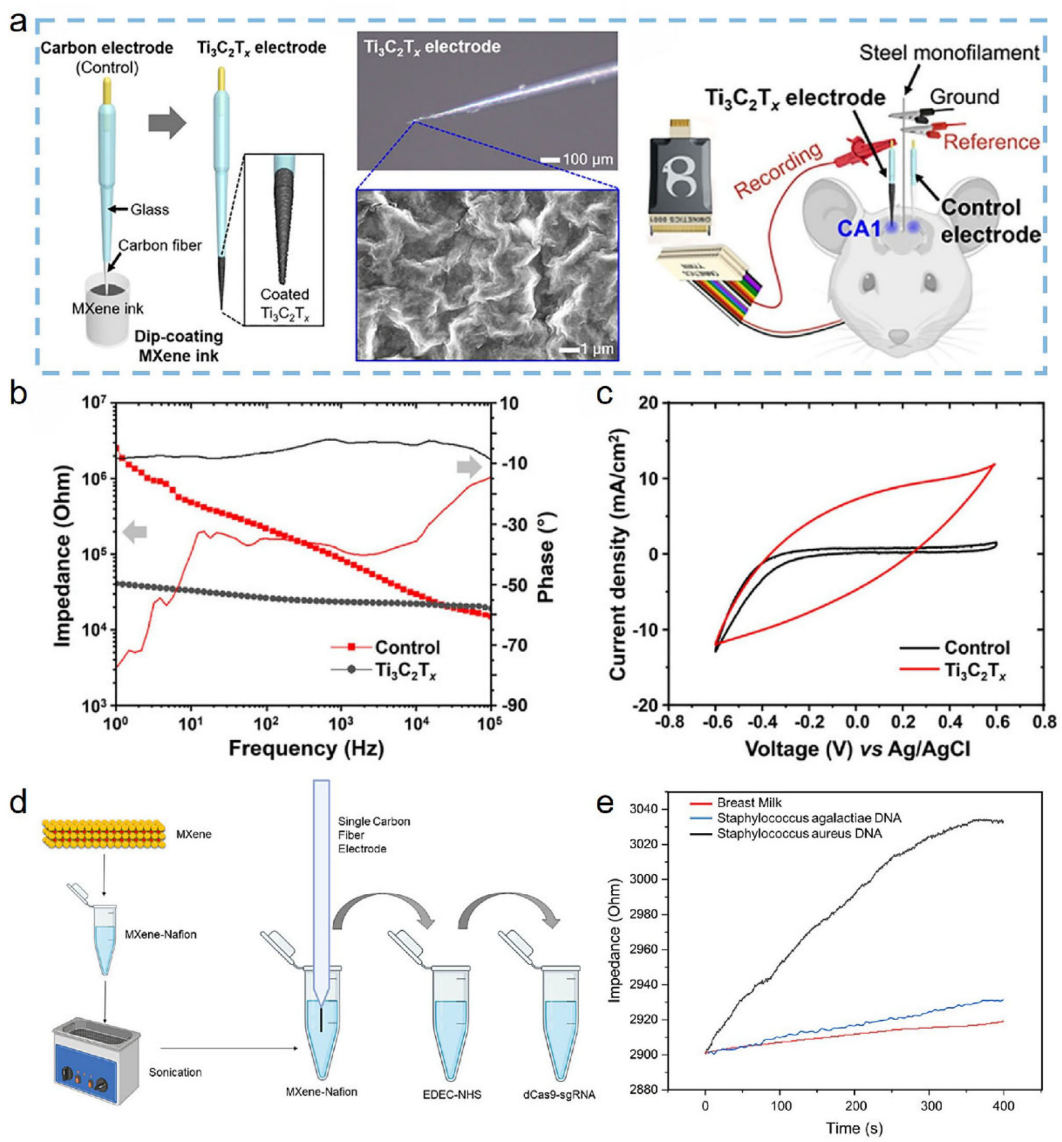
(a) Schematic diagram of Ti_3_C_2_T_x_ MXene‑modified CF brain electrode and the in vivo recording setup for electrophysiological brain signal recording. (b) Impedance spectra and corresponding phase angles and (c) cyclic voltammetry curves of control CF and Ti_3_C_2_T_x_ MXene‑modified CF electrodes. (a–c) Reproduced under terms of the CC‐BY license [156]. Copyright 2025, Wiley. (d) Schematic diagram of Ti_3_C_2_T_x_ MXene‑modified CF electrode preparation for pathogenic DNA detection in breast milk samples. (e) Chronoimpedimetric detection of biosensor. (d, e) Reproduced under terms of the CC‐BY license [157]. Copyright 2024, American Chemical Society.

Ti_3_C_2_T_x_ MXene‐modified CF can significantly reduce the interfacial impedance of CF electrodes, improve the signal‐to‐noise ratio, and stimulation efficiency. Meanwhile, its flexibility ensures good compatibility with neural tissues and reduces inflammatory responses after long‐term implantation, making it an excellent candidate for bioelectrode materials [155]. For instance, Kondrataviciute et al. [156]. prepared Ti_3_C_2_T_x_ MXene‐modified CF electrodes for deep brain recording (Figure 15a). Compared to naked CF electrodes, Ti_3_C_2_T_x_ MXene‐modified electrodes exhibited lower impedance, higher signal fidelity, and excellent biocompatibility, pointing toward advanced neural interfaces and microelectrode arrays for brain‐machine interfacing or neural stimulation/recording (Figure 15b,c).

In addition, the ongoing development of Ti_3_C_2_T_x_ MXene‐modified CFs for intelligent sensing is expected to extend to biosensing as well, offering broad potential. For example, Ertuğrul et al. [157]. fabricated biosensors using Ti_3_C_2_T_x_ MXene‐modified CF electrodes for the rapid, sensitive, and selective detection of Staphylococcus aureus DNA in breast milk (Figure 15d,e), achieving a linear detection range from 50 to 6000 fM and a detection limit of 14.5 fM. Recently, such biosensors have shown high sensitivity and real‐time monitoring for diverse analytes, including disease markers, neurotransmitters, and therapeutic drugs, paving the way for new‐generation biomedical devices and advancing clinical diagnostics and precision medicine [130, 158].

In summary, Ti_3_C_2_T_x_ MXene‐modified CFs provide an innovative materials platform for biomedical device development. To successfully translate these promises from laboratory research into practical clinical applications, a concerted focus on several critical pathways is essential. First, establishing comprehensive, standardized biosafety evaluation systems is paramount, requiring rigorous long‐term in vivo studies to assess biocompatibility, degradation profiles, and systemic immune responses under physiological conditions. Second, research must refine and tailor surface functionalization strategies to move beyond general biocompatibility toward enabling specific biomolecular recognition, targeted drug delivery, or directed cellular interactions. Thirdly, the development of robust, scalable, and reproducible manufacturing processes compatible with clinical and regulatory standards is crucial for commercialization. Addressing these interconnected challenges through interdisciplinary collaboration will be key to accelerating the clinical adoption of these multifunctional composites for applications such as neural interfaces, bone regeneration scaffolds, and implantable biosensors.

## 4 Challenges and Future Prospects

Despite the immense application potential of Ti_3_C_2_T_x_ MXene‐modified CFs in multifunctional composites, energy storage, and catalysis, the transition from laboratory research to practical deployment remains beset by numerous challenges. These hurdles mainly encompass material stability, scalable fabrication, performance optimization, and mechanistic understanding. Future development will require breakthroughs in material design, processing technology, application expansion, and interdisciplinary integration.

### 4.1 Major Challenges

A prominent issue is the oxidative degradation of Ti_3_C_2_T_x_ MXene. Under conditions of high humidity, oxygen‐rich atmospheres, or elevated temperatures, Ti_3_C_2_T_x_ MXene readily oxidizes to corresponding metal oxides (such as TiO_2_), resulting in a sharp decline in its outstanding electrical conductivity and catalytic activity [34, 159]. This drawback critically restricts the long‐term reliability of Ti_3_C_2_T_x_ MXene‐modified CFs, particularly in harsh environments. In addition, the interfacial bonding between Ti_3_C_2_T_x_ MXene and CF is another key concern. Most current studies rely on weak physical interactions (e.g., van der Waals forces, hydrogen bonding) for Ti_3_C_2_T_x_ MXene‐CF integration, which are prone to failure under prolonged stress or chemical corrosion [160]. Although covalent bonding can greatly improve interfacial stability, such methods usually require complex or time‐consuming surface pretreatments (e.g., plasma treatment, functional group grafting), increasing process complexity and cost [161, 162].

Current synthesis and modification methods for Ti_3_C_2_T_x_ MXene face significant limitations. Its typical preparation via HF etching uses highly toxic and corrosive reagents, placing strict demands on equipment and posing significant environmental hazards, which greatly impede large‐scale production [163, 164]. While fluoride‐free techniques (such as electrochemical or molten salt etching) are emerging, they still present challenges in quality control, process stability, and product consistency. The fabrication processes for Ti_3_C_2_T_x_ MXene‐modified CFs (e.g., EPD method, self‐assembly, chemical grafting) often require precise control of parameters (such as pH, concentration, voltage), complicating continuous processing and resulting in low manufacturing efficiency. Moreover, cost remains a major barrier to commercialization, as high‐grade MXene precursors, energy consumption during modification, and environmental management all contribute to relatively high expenses [165].

Realizing balanced and integrated multifunctionality in Ti_3_C_2_T_x_ MXene‐modified CFs represents a highly complex challenge. Although increasing Ti_3_C_2_T_x_ MXene content typically enhances the electrical conductivity of the composites as well as functional properties such as EMI shielding and energy storage, excessive loading can easily induce nanosheet agglomeration and interfacial stress concentration, thereby disrupting load transfer pathways and ultimately compromising the material's mechanical integrity and long‐term durability [70, 100]. A more profound conflict lies in the fact that the core material properties required for integrating different functions are often mutually contradictory. For instance, to achieve excellent EMI shielding or energy storage performance, high electrical conductivity is sought, which favors increasing Ti_3_C_2_T_x_ MXene loading and strengthening interfacial electronic coupling, but may simultaneously lead to a reduction in material toughness or an increase in brittleness. Conversely, if structural strength and damage tolerance are the primary goals, a toughened interface needs to be designed, which may hinder the formation of an ideal, efficient conductive pathway. Similarly, in applications such as wearable sensing or flexible devices, the material must maintain a certain degree of structural stiffness to bear loads while also possessing sufficient flexibility to accommodate deformation, constituting a fundamental design dilemma. Furthermore, the randomness in the bonding modes between Ti_3_C_2_T_x_ MXene and CFs, along with the difficulty in precisely controlling their spatial arrangement at the nanoscale, remains a critical bottleneck that constrains the predictability and reproducibility of material performance as well as the full release of functionality.

A deep understanding of the mechanisms underlying the property enhancement and failure behavior of Ti_3_C_2_T_x_ MXene‐modified CF composites is still lacking. Quantitative elucidation of the key interfacial reinforcement mechanisms, such as mechanical interlocking, chemical bonding, and electrostatic interaction, remains underexplored. Studies on the evolution and failure mechanisms of composite properties under long‐term service, especially in harsh environments (e.g., humidity, chemical exposure), are nearly absent. There is also a shortage of in situ characterization data under realistic working conditions (e.g., load, deformation, and high temperature), which impedes the ability to accurately correlate material structure with performance.

### 4.2 Future Prospects

Future research should strive to address the above‐mentioned challenges and promote the practical application of Ti_3_C_2_T_x_ MXene‐modified CFs through interdisciplinary integration. In terms of material design, the development of composite systems combining Ti_3_C_2_T_x_ MXene with high‐performance nanomaterials (such as metal nanoparticles, MOFs, CNTs, etc.) represents a promising direction. The synergistic effects of such heterostructures can greatly broaden the application scenarios of Ti_3_C_2_T_x_ MXene‐modified CFs. Tailoring the structural features and surface terminal groups of Ti_3_C_2_T_x_ MXene is another key strategy, enabling precise control of its electrical, chemical, and mechanical properties, thus optimizing the performance of Ti_3_C_2_T_x_ MXene‐modified CFs for various specialized applications. Additionally, the incorporation of bio‐inspired structural designs (e.g., nacre‐like architectures, mussel‐inspired multiscale architectures) offers the potential for achieving simultaneously high strength, toughness, and multifunctionality.

For fabrication processes, the development of green, efficient, and low‐cost Ti_3_C_2_T_x_ MXene synthesis techniques is a prerequisite for large‐scale application. Fluoride‐free etching methods (such as electrochemical etching, molten salt etching, etc.) should be further optimized to improve yield and product quality. The development of mature, continuous production processes (such as roll‐to‐roll coating or online deposition) will be critical for high‐throughput manufacturing of Ti_3_C_2_T_x_ MXene‐modified CFs. This necessitates coordinated consideration of feasibility, cost, and environmental impact to advance commercialization. Furthermore, the integration of artificial intelligence and machine learning into the fabrication process is expected to facilitate rapid optimization of process parameters, achieve precise control over the distribution, orientation, and coverage of Ti_3_C_2_T_x_ MXene on CFs, and substantially improve productivity and industry development.

In terms of stability enhancement, effective anti‐oxidation strategies for Ti_3_C_2_T_x_ MXene are required. Surface passivation, encapsulation, or compounding with more stable materials are potential solutions. Additionally, the establishment of accelerated aging tests and long‐term performance prediction models will be essential for evaluating the reliability of Ti_3_C_2_T_x_ MXene‐modified CFs under extreme conditions and for supporting their deployment in specific environments.

For the conflicts in material performance, future research should move beyond simple compromise solutions and instead explore strategies such as establishing an application‐oriented functional priority hierarchy, which involves clearly defining the primary and secondary functions of the composite for specific applications to guide targeted optimization; developing dynamically tunable interfaces that utilize stimuli‐responsive polymers or dynamic covalent bonds to achieve reversible adjustment of interfacial properties in response to external fields (e.g., temperature, stress, electric field), thereby adapting to different functional requirements under varying working conditions; and constructing spatially graded or heterogeneous microstructures, which involve spatially controlling the distribution, orientation, or chemical state of Ti_3_C_2_T_x_ MXene to impart differentiated properties to different regions of the fiber, realizing “zoned functionalization” and thereby integrating seemingly contradictory properties at a macroscopic scale.

For mechanistic understanding, it is essential to move beyond the traditional linear approach of “simulate first, characterize next, and analyze afterward.” Instead, the field should transition toward an intelligent research framework centered on “digital twins” and driven by high‑throughput experimentation. In essence, this involves creating a highly realistic virtual replica of the physical material system within a computational environment. High‑throughput experiments are then employed to rapidly generate large‑scale data for training and refining this digital model, thereby establishing a closed loop of data‑driven modeling, model‑guided experimentation, and experimental feedback optimization. This iterative cycle will shift the research mindset from providing post‑factum mechanistic explanations of observed phenomena toward enabling dynamic prediction and proactive design of material properties. As a result, it will foster a paradigm transition from localized performance optimization to system‑level synergistic enhancement. Such a deep integration of intelligent technology and experimental science will not only yield unprecedented insights into the cooperative reinforcement mechanisms between Ti_3_C_2_T_x_ MXene and CF, but will also play a solid and innovative theoretical foundation for the rational design of materials tailored to future complex application scenarios.

Regarding application expansion, Ti_3_C_2_T_x_ MXene‐modified CFs can be further developed toward intelligent and multifunctionally integrated materials. This includes the creation of structure‐energy integrated materials (e.g., structural supercapacitors, structural batteries), smart sensing composites with self‐monitoring or adaptive capabilities. Similarly, environment‐responsive smart materials (thermo‐, pH‐, photo‐responsive) can be realized by grafting stimuli‐sensitive polymers onto the conductive CF/MXene network. Furthermore, the application of Ti_3_C_2_T_x_ MXene‐modified CFs in areas such as biomass conversion, photoelectrocatalysis, optical sensing, and biomedicine represents a highly promising research direction.

Finally, interdisciplinary collaboration and the integration of academia and industry are vital drivers of commercial implementation. Stronger cooperation among materials scientists, chemists, engineers, and industrial partners is required to establish an efficient pathway from fundamental research to industrialization. At the same time, the gradual establishment of standardized preparation methods, performance evaluation systems, lifetime assessment protocols, and green recycling schemes will provide critical technical support for industrial deployment.

## 5 Conclusion

In conclusion, Ti_3_C_2_T_x_ MXene‐modified CFs signify a transformative leap from singular structural materials to integrated multifunctional platforms. This evolution is underpinned by advanced fabrication strategies such as controlled coating, self‐assembly, EPD, chemical grafting, and blending‐spinning, which enable precise interfacial engineering and holistic integration of Ti_3_C_2_T_x_ MXene's exceptional electrical, thermal, and electrochemical properties. These composites demonstrate synergistic performance across diverse fields, including enhanced interfacial reinforcement, superior EMI shielding, efficient energy storage, and emerging applications in smart sensing, thermal management, catalysis, and biomedicine.

Notably, the realization of this structure‐function paradigm hinges on addressing persistent challenges such as Ti_3_C_2_T_x_ MXene's oxidative instability, scalable and eco‐friendly synthesis, and the pursuit of robust interfacial bonding. Future advancements also call for innovative material designs like fluoride‐free etching routes, and covalent bonding techniques compatible with industrial‐scale production. Furthermore, interdisciplinary collaboration will be essential to deepen the understanding of interfacial mechanisms and to guide the rational design of next‐generation composites.

Ultimately, this integration of CF's structural robustness with Ti_3_C_2_T_x_ MXene's multifunctional activity opens pathways for lightweight, adaptive, and intelligent material systems. By bridging the gap between structural components and functional devices, Ti_3_C_2_T_x_ MXene‐modified CFs hold substantial potential to transform industries ranging from aerospace and transportation to wearable electronics and sustainable energy, marking a significant step toward future high‐performance, multi‐purpose engineering materials.

## Author Contributions

**Hongshuo Cao**: conceptualization, investigation, visualization, and writing – original draft. **Yue Xing**: conceptualization, visualization, and supervision. **Jiangman Sun**: conceptualization, visualization, and supervision. **Yanhong Tian**: conceptualization and supervision. **Yangyang Gao**: conceptualization and supervision. **Xuejun Zhang**: conceptualization, supervision, project administration, writing – review and editing. **Xiubing Liang**: conceptualization and supervision.

## Conflicts of Interest

The authors declare no conflict of interest.

## Data Availability

The authors have nothing to report.
